# Reversibility and therapeutic feasibility of *DNM1L*-associated neurodevelopmental disorders

**DOI:** 10.1038/s12276-026-01660-z

**Published:** 2026-03-05

**Authors:** Ki Hurn So, Se Hee Kim, Shinyoung Jang, Hyeun Deok Sang, Eun-Jin Yun, Hee-Jung Choi, Jong-Hee Chae, Seung Tae Baek

**Affiliations:** 1 Department of Life Sciences, Pohang University of Science and Technology, Pohang, Republic of Korea; 2 Pediatric Neurology, Department of Pediatrics, Epilepsy Research Institute, Severance Children’s Hospital, Yonsei University College of Medicine, Seoul, Republic of Korea; 3 School of Biological Sciences, Seoul National University, Seoul, Republic of Korea; 4 Department of Genomic Medicine, Seoul National University Hospital, Seoul, Republic of Korea; 5 Department of Pediatrics, Seoul National University College of Medicine, Seoul, Republic of Korea

**Keywords:** Paediatric neurological disorders, Disease model

## Abstract

*DNM1L*-associated encephalopathy is a neurological disorder with a broad spectrum of symptoms associated with mutations in the *DNM1L* gene. Treatment primarily aims to alleviate symptoms, which is mostly ineffective as the underlying neuropathology is not well understood. Moreover, the progression and reversibility of the molecular pathology across the key developmental and postnatal stages have not been well characterized, which is crucial for identifying the therapeutic window and formulating effective treatment strategies. Here we demonstrated that the expression of *DNM1L* variants in developing mouse brains caused severe neuronal loss pronounced at the early postnatal stage. Using a human stem cell model with chemogenetic control of *DNM1L*, we elucidated the neurodevelopmental stage-specific reversibility of transcriptional changes caused by *DNM1L* dysfunction. Noticeably, more than 75% of the transcriptional landscape associated with pathology can be restored even in differentiated neurons. We validate that a feasible therapeutic strategy targeting one of the reversible pathways, mitochondrial biogenesis, prevents neurodegeneration, suggesting the potential for effective postnatal clinical intervention in *DNM1L*-associated disorders.

## DNM1L variants reveal potential for reversing neurodegeneration

*DNM1L*-associated encephalopathy is a rare brain disorder caused by mutations in the *DNM1L* gene, which affects the DRP1 protein. DRP1 is crucial for cell functions such as energy production and cell death. Mutations in *DNM1L* can lead to severe symptoms, including developmental delays and epilepsy. Despite advances in diagnosis, the exact brain changes due to these mutations are not well understood. Researchers studied these changes using mouse and human cell models. They found that harmful *DNM1L* mutations cause neuron loss after birth in mice. They also identified specific gene activity changes in human nerve cells with the *DNM1L* dysfunction. By boosting mitochondrial biogenesis, they improved brain features in affected mice. This study used genetic analysis and cell culture techniques to explore these effects. The results suggest that enhancing mitochondrial function could help treat *DNM1L*-associated encephalopathy.

This summary was initially drafted using artificial intelligence, then revised and fact-checked by the author.

## Introduction

*DNM1L*-associated encephalopathy (DAE) is a rare neurological disorder associated with de novo monoallelic and biallelic variants in the *DNM1L* gene encoding the DRP1 protein^1–14^. DRP1 is a guanosine triphosphatase (GTPase) protein involved in mitochondrial and peroxisomal fission, which is essential for maintaining various cellular processes such as metabolism, apoptosis and differentiation^15^. Patient fibroblasts and peripheral blood mononuclear cells showed hyperfused mitochondria and elongated peroxisomes, indicating the dominant-negative or loss-of-function effects of *DNM1L* mutations^1,13,14,16,17^. DRP1 is indispensable for neurodevelopment in mammals, as indicated by neonatal lethality due to cerebellar degeneration in neuron-specific DRP1 knockout (KO) mice^18,19^. Accordingly, mutations or dysregulation of DRP1 have been implicated in several neurodegenerative and neurodevelopmental diseases^1,8,14,20–22^. Despite the advances in diagnosing DAE, the neuropathological mechanisms of *DNM1L* mutations remain poorly defined.

The phenotypic features of DAE include developmental regression, microcephaly, refractory epilepsy, hypotonia, cerebral and optic atrophy and even neonatal death in severe cases^1–14,23^. Literature reviews reported that most patients showed abnormal brain magnetic resonance imaging (MRI), represented by cerebral atrophy and corpus callosum (CC) thinning, with a median age of onset being 6 months^12,14,24^. Currently, there is no effective cure owing to the complexity and diversity of neurological characteristics in patients. Treatments such as anti-epileptic drugs and vitamin cocktail therapy have been used to alleviate symptoms such as seizures, with limited clinical benefits observed^4,5,10,12,23^. Moreover, the progression of DAE pathology at the molecular level across the key developmental and postnatal stages has not been well characterized, and its reversibility, which provides insights into optimally timed therapeutic intervention, remains poorly understood.

Here, we explored the pathology, reversibility and treatment potential of DAE using both mouse and human neuronal cell models. Our findings demonstrate that pathogenic *DNM1L* variants lead to postnatal neuronal loss in the DAE mouse model. We also characterized stage-specific and reversible transcriptional landscapes underlying *DNM1L* dysfunction in differentiated human neuronal cells. Treatments enhancing mitochondrial biogenesis during the prenatal and postnatal stages ameliorated the neuropathological features of DAE.

## Materials and methods

### Genetic analysis of patients with DAE

The study received approval from Severance Hospital’s Institutional Review Board at Yonsei University Health System (approval no. 4-2020-0331). All participants and/or their caregivers provided written informed consent for genetic testing.

Genomic DNA was extracted from the peripheral blood of both patients using the QIAamp DNA Mini Kit (QIAGEN), measured using the Qubit HS dsDNA kit (Invitrogen), and underwent clinical exome sequencing on the NovaSeq 6000 System (Illumina). Raw sequence data were aligned to the GRCh37 (hg19) reference genome, and protein-coding variants were filtered by mapping quality score (≥ 80) and coverage depth (≥ 20×). *DNM1L* c.1247T>C and c.1949T>G were identified as the primary candidate mutations in the respective patients.

### In silico mutation impact analysis

To assess the impact of mutations on the DRP1 tetramer structure (Protein Data Bank (PDB), 4BEJ), FoldX5 was used to predict ΔΔ*G* values. Calculations were performed using the BuildModel option.

### DNA constructs

The pCAG-IRES-green fluorescent protein (GFP) (pCIG) vector and PiggyBac Transposon vector (System Biosciences, #PB513B-1) were used for cloning human *DNM1L*. Variants (*DNM1L*^*E2A*^, *DNM1L*^*G350R*^, *DNM1L*^*L416P*^ and *DNM1L*^*L650R*^) were generated by block PCR-based mutagenesis. To establish the *DNM1L* Tet-off system on the basis of the pCW57.1-MAT2A lentiviral vector (Addgene, #100521), we replaced the *MAT2A* sequence with *DNM1L* wild type complementary DNA under the TRE promoter and substituted the blasticidin sequence with GFP for fluorescence-based selection. pCMV-VSV-g (Addgene, #8454) and pCMV-delta 8.91 plasmids were used for lentivirus packaging. Puromycin and GFP sequences were cloned into the CRISPR–Cas9 system pX330 (Addgene, #42230) to generate a pX330-puro-GFP vector. Single-guide (sg)RNA targeting endogenous human *DNM1L* exon 1 was designed with Synthego online tool (Supplementary Table 2). Mouse *PPARGC1A* cDNA was PCR-amplified from the NIH3T3 cDNA library and cloned into the pCAG-IRES-DsRed vector. pcDNA5-MTS-TagBFP-P2AT2A-EGFP-NLS-P2AT2A-mCherry-PTS1 (Addgene, #87813) was used for organelle visualization. pRP-CAG-hyPBase was designed and purchased from VectorBuilder. All clones were verified by sequencing.

### In utero electroporation and tissue processing

All animal procedures followed the guidelines of the Pohang University of Science and Technology Institutional Animal Care and Use Committee (IACUC) (POSTECH-2019-0071; POSTECH-2020-0077). C57/BL6 mice from Hyochang Science were used for all animal experiments. For timed pregnancy, the noon of checked vaginal plug was considered embryonic day (E) 0.5. Surgery and electroporation were performed as described in Kim et al.^25^. In brief, pregnant mice were anesthetized with isoflurane (induction: 3%, surgery: 2%, Hana Pharm Corporation, #657801261). Endotoxin-free plasmids (1–2 μg/μl) with 0.1% Fast Green (Sigma, #F7252) were injected into one of the lateral ventricles of embryos. Electric pulses were delivered using ECM 830 Square Wave Electroporation System (BTX Harvard Apparatus) with parameters adjusted according to embryonic age: five pulses of 35 V for 50 ms with 950-ms intervals at E13.5 for prenatal apoptosis analysis, four pulses of 45 V for 50 ms with 500-ms intervals at E14.5 for prenatal proliferation and migration analysis and five pulses of 45 V for 50 ms with 500-ms intervals at E15.5 for postnatal analysis. Platinum Tweezertrode (5 mm in diameter, BTX Harvard Apparatus, #45-0489) was used to target excitatory neural progenitors in the ventricular zone of the cortex.

For tissue collection, brain samples before postnatal day (P) 7 were collected via decapitation, while pregnant dams were killed via cervical dislocation after isoflurane-mediated anesthesia. For frozen embedding, embryonic brains were isolated at designated time points (E15.5 for prenatal apoptosis and proliferation analysis; E18.5 for BrdU chasing and migration analysis), fixed with 4% paraformaldehyde (PFA) in phosphate-buffered saline (PBS) overnight at 4 °C, washed three times with PBS for 15 min each and cryoprotected with 30% sucrose in PBS overnight at 4 °C. For perinatal time course analysis, brains were collected at the early (P2), middle (P5) and late stages (P7) of the first postnatal week. Following fixation with 4% PFA in PBS overnight at 4 °C, cryoprotection was conducted sequentially overnight with 15% and 30% sucrose in PBS. For P21 brains, mice were anesthetized with isoflurane and perfused intracardially with PBS, followed by 4% PFA in PBS. Tissues were fixed with 4% PFA overnight and subsequently dehydrated in a series of sucrose solutions (10%, 20% and 30% in PBS). All samples were embedded in optimal cutting temperature compound (Tissue-Tek, #HIO-0051) and cryosectioned at 12 μm for slide-mounted immunostaining, 35 μm for floating immunostaining and confocal imaging or 100 μm for axon branching visualization. Samples in each condition were collected from at least three different litters to avoid batch effect, and the number of animals used is specified in each figure legend. We ensured that samples in the same condition had comparable tissue quality, transfected regions and overall transfection efficiency to minimize sample-to-sample variability during analysis.

### Primary mouse cortical neuron culture

Embryo brains at E15.5 were injected with endotoxin-free plasmids and electroporated to target cortical excitatory neural progenitors. The neocortex was dissected from embryos and serially washed in 1× Hanks’ Balanced Salt Solution (Gibco, #14175095) with 20 mM HEPES (Gibco, #15630-106) to minimize contamination. The tissue was dissociated with papain (Worthington Biochemical, #LK003176) solution mixed with 10 μg/ml DNase I (Sigma-Aldrich, #DN25-10MG) at 37 °C for 10 min, then triturated about 15–20 times to obtain a single-cell suspension. A total of 10^5^ cells per 300 μl were plated on cover glass coated with poly-D-lysine (Gibo, #A3890401). After 2 h of incubation, Neurobasal medium (Gibco, #21103049) supplemented with 1× B27 (Gibco, #17504044), 1× GlutaMAX (Gibco, #35050-061), 100 U/ml penicillin–streptomycin (Gibco, #15140148) and 2.5% fetal bovine serum (FBS) (Corning, #35-015-CV) was added. On days in vitro (DIV) 5, cells were cultured in the medium without FBS. The highest dose of bezafibrate with minimal toxicity (400 μM) treatment was used following the references, and the medium was exchanged daily during bezafibrate treatment^26,27^. Cell viability was analyzed by live imaging of GFP-positive cells from DIV 4 to 10 at 37 °C in a 5% CO_2_ atmosphere. All cells were tested negative for mycoplasma via routine PCR-based amplification.

### Chemicals and reagents

Mitotracker Red CMXRos (Invitrogen, #M7512) was used to label mitochondria in human neural progenitor cells (NPCs) according to the manufacturer’s instructions (200 nM for 15 min). BrdU (Sigma, #B5002) dissolved in saline with 7 mM NaOH was administrated maternally (100 mg/g body weight) by intraperitoneal injection. Doxycycline (DOX) (Sigma, #D9891) stock was prepared in dimethylsulfoxide (DMSO) (Sigma, #D2650). Bezafibrate (Sigma, #B7273) stock was prepared in DMSO (50 mg/ml) and diluted with vehicle (corn oil, Sigma, #C8267) before injection. Bezafibrate (30 mg/kg body weight) was intraperitoneally injected into neonatal mice on the basis of a previous study^28^.

### Human cell lines culture

H9 (WA09, WiCell) human embryonic stem (ES) cells were cultured on Matrigel (Corning, #354277) with daily exchange of mTeSR1 (Stem Cell Technologies, #85850) medium containing antibiotics (100 U/ml penicillin–streptomycin). Human ES cells were maintained by manually removing differentiated colonies and passaged about every 5–6 days by Gentle Cell Dissociation Reagent (Stem Cell Technologies, #100-0485). HEK293T cells were cultured in DMEM supplemented with 10% FBS and antibiotics. NPCs were generated from human ES cells using the StemDiff SMADi Neural Induction Kit (Stemcell Technologies, #08581). After three passages, NPCs were plated onto Matrigel (Corning, #354234) and cultured in NBF media (DMEM-F/12 supplement with 0.5X B27, 0.5X N2 (Gibco, #17502001), antibiotics and 20 ng/ml bFGF (Stemcell Technologies, #78003)). NPCs were exchanged with fresh NBF media every other day and passaged by Accutase (Merck, #SCR005) when it reached 100% confluency. For spontaneous neural differentiation, NPCs were cultured in NB media (NBF media without bFGF), which was replaced every 2 days. All cell lines were maintained at 37 °C in a 5% CO_2_ atmosphere and tested negative for mycoplasma via routine PCR-based amplification.

### Transfection and lentivirus transduction

NPCs were transfected using Lipofectamine 3000 (Invitrogen, #L3000015) and Opti-MEM (Gibco, #31985070). The total DNA quantities were adjusted according to the culture size, following the manufacturer’s instructions. For lentiviral production, the pCW57.1-*DNM1L* lentiviral vector was transfected into HEK293T cells together with packaging plasmids using Lipofectamine 3000. Lentivirus-containing mTeSR1 media were collected after 48 and 72 h and filtered using 0.45-μm filters (Pall Corporation, #4614). Human ES cells were transduced with lentivirus-containing mTeSR1 media for 24 h. At 72 h after transduction, human ES cells were sorted for GFP expression using Moflo Astrios (Beckman Coulter).

### Genome editing of human ES cells

Endogenous *DNM1L* KO human ES cells were generated using Human Stem Cell Nucleofector Kit 2 following the manufacturer’s instructions (Lonza, #VPH-5002). In brief, human ES cells were collected as single cells using Accutase and mixed with the pX330-puro-GFP vector containing *DNM1L* targeting sgRNA. Electroporation was performed using the B-016 program in the NucleofectorTM II/2b Device (Lonza, #LOAAB-1001). Electroporated human ES cells were replated in mTeSR1 media with Y-27632 (10 μM, Stemcell Technologies, #72304) for 24 h. Puromycin (0.5 μg/ml, Gibco, A11138-03) selection was performed for 48 h 2 days after electroporation. Single cell-derived human ES cell clones were isolated and maintained individually. Genomic DNA was extracted using Wizard SV Genomic DNA Purification System (Promega, #A2360), and the PCR-amplified target region was identified by Sanger sequencing. Indels were detected by CRISP-ID^29^.

### Flow cytometry analysis

NPCs were cultured with B27 deprivation for 2 days to minimize potential protective effects by antioxidants contained in B27. NPCs were then treated with 150 μM H_2_O_2_ in Hanks’ Balanced Salt Solution for 3 h to induce cell death. The concentration of H_2_O_2_ was selected by screening a dose range (about 0–200 μM) that induces cell death without causing excessive cell death across all conditions^30^. Cells were incubated with Annexin V–CF Blue and 7-AAD for 15 min in the dark, following the manufacturer’s protocol. Apoptotic and necrotic populations were analyzed with CytoFLEX S (Beckman Coulter). A minimum of 50,000 cells were used for analysis.

### Neurosphere formation assay

NPCs were collected with Accutase and plated into noncoated low-attachment plates with NBF media. Neurospheres were formed with 24 h of gentle shaking (50 rpm) in the incubator and cultured for an additional 24 h in NBF media. Neurospheres were collected and replenished with fresh NB media every 2 days for further differentiation. For the KO induction in neurospheres, 5 ng/μl DOX was used throughout the process. After 1 week of differentiation, neurospheres were fixed with 4% PFA, washed with PBS and processed for downstream immunostaining.

### Immunostaining

For immunohistochemistry, sections were air-dried, rinsed with PBS, permeabilized with 0.1% Triton X-100 in PBS and blocked with CAS-Block histochemical reagent (Invitrogen, #008120). For BrdU staining, the antigen retrieval was performed at 95 °C for 15 min with a citrate buffer of pH 6.0 before permeabilization. Sections were then immunostained with primary antibodies overnight at 4 °C. After washing three times with PBS for 10 min each, the appropriate secondary antibodies were applied for 30 min at room temperature. Nuclei were stained with 4,6-diamidino-2-phenylindole (DAPI) (Invitrogen, #D1306). Tissue sections were mounted in 80% glycerol in PBS, and 100-μm-thick sections were mounted with Aqua-Poly/Mount (Polysciences, #18606). For immunocytochemistry, processes were mostly identical to immunohistochemistry with minor modifications: coverslips or neurospheres were placed on slide glasses and mounted with 80% glycerol in PBS. The primary antibodies used were as follows: PMP70 (Invitrogen, #PA1-650, 1:500), Ctip2 (Abcam, #ab18465, 1:500), Satb2 (Abcam #ab51502, 1:400), Ki67 (Abcam, ab15580, 1:500), cleaved caspase 3 (CC3) (Cell Signaling Technologies, #9661, 1:500), phospho-histone H3 (pHH3) (Cell Signaling Technologies, #9706, 1:500), BrdU (BD Biosciences, #347580, 1:100) and GFP (Rockland, #600-106-215, 1:100).

### Protein extraction and western blot

Whole proteins were extracted upon cell lysis with ice-cold RIPA buffer (Rockland, #RKMB-030-0050) containing phosphatase and protease inhibitors (Quartett, #QTPPI1041 and #QTPPI1015). Proteins were separated by electrophoresis on 4–15% Mini-PROTEAN precast gels (Bio-Rad, #4561086), transferred onto nitrocellulose membranes (Bio-Rad, #1704159), blocked with 5% skimmed milk (w/v) in PBST (0.1% Tween-20 in 1× PBS) for 1 h at room temperature and treated with primary antibodies: DRP1 (Abcam, #ab56788, 1:1000) and β-actin (Cell Signaling Technologies, #4970, 1:8000). After incubation with respective secondary antibodies coupled with horseradish peroxidase, antibody binding was detected using enhanced chemiluminescence substrate (Enzynomics, #EOE001S). Chemiluminescence signals were imaged using LAS4000 (GE Lifescience).

### Experimental design for RNA sequencing in neuronal cells

To turn off gene expression in the Tet-Off system, 5 ng/μl DOX treatment was administered for 10 days during two passages at the NPC stage and for 3 weeks at the neuron stage for subsequent bulk RNA sequencing. Control samples were treated with the same volume of DMSO. For rescue schemes, NPCs treated with 5 ng/μl DOX for 10 days during two passages were prepared. For developmental rescue (NPC-Rescue), DOX-treated NPCs were withdrawn from DOX, washed with Dulbecco’s PBS (DPBS) twice, replenished with NB media and maintained for 4 weeks. For neuronal rescue (NEU-Rescue), DOX-treated NPCs were washed with DPBS twice, cultured in NB media with 5 pg/μl DOX for 2 weeks and then switched to fresh NB media without DOX for another 2 weeks. The lower DOX concentration (5 pg/μl) was used for reliable re-expression in differentiated neurons. All conditions were prepared in triplicate.

### RNA sequencing and quantitative PCR with reverse transcription

Total RNA was prepared with the Quick RNA mini-prep kit (Zymo Research, #R1054). The library was constructed with TruSeq stranded messenger RNA LT sample prep kit (Illumina) and sequenced by HiSeq2000. RNA was reverse transcribed with the LunaScript (NEB, #E3010L). The quantitative PCR (qPCR) analysis was performed using Luna Universal SYBR (NEB, #M3003L) on the MIC qPCR Cycler (BMS). The primers were retrieved using National Center for Biotechnology Information (NCBI) primer blast. *RPLP0* was used as a normalization control. The primers used are presented in Supplementary Table 2.

### Transcriptomic analysis

The quality of sequencing reads was measured using FastQC, and adapter sequences were removed with Trimmomatic. Reads were aligned to the GRCh38_NCBI_109 reference genome using the Bowtie2 aligner. StringTie was then used to assemble the aligned reads into known genes or transcripts and to calculate read counts, fragments per kilobase of transcript per million mapped reads, and transcripts per million. Differentially expressed genes (DEGs) were identified using the DESeq2 package in R, with genes having a total read count <13 being excluded. Genes with an absolute fold change >2 and an adjusted *P* value threshold <0.05 between conditions were defined as DEGs. Gene Ontology (GO) enrichment analyses were performed using the EnrichR and SynGO online tools. Nonspecific or irrelevant terms related to neuronal progenitor cells or neurons were excluded from the bar plot visualizations. Heat maps were generated using *Z* scores obtained through the variance stabilizing transformation function in DESeq2. For meta-analysis, the gene expression data were corrected for possible batch effects by applying linear modeling in the limma package. Control samples selected from each batch were used to create batch-corrected heat maps. For the prediction of the upstream regulator, the ChEA3 online tool was used. A rank–rank hypergeometric overlap (RRHO) test was performed in the RRHO2 package in R. Full differential expression lists were ranked by the stat value of the Wald test (log_2_ fold change divided by the standard error of log_2_ fold change) from DESeq2. RRHO2 heat maps visualize degrees of overlap of genes changed in the same and opposite directions between two datasets.

To assess the transcriptional reversibility of pathways disrupted in DNM1L^OFF^, we developed a quantitative reversibility score incorporating two essential parameters: the percentage of reversed genes (P) and the initial perturbation magnitude (D). Gene expression was normalized using the variance-stabilizing transformation of DESeq2. Genes were defined as ‘reversed’ if their expression in NPC-Rescue or NEU-Rescue conditions moved toward control levels after significant alteration in the KO condition (using thresholds of log_2_ fold change >1 and adjusted *P* < 0.05). For each Reactome database pathway, we calculated *P* as the proportion of reversed genes relative to total pathway genes, while *D* represented the average absolute expression difference between KO and control samples, indicating perturbation intensity. The pathways were displayed in scatter plots with *P* on the *x* axis and *D* on the *y* axis, with functionally relevant pathways highlighted for clarity.

### Imaging and quantifications

Immunostained slides were imaged using a Nikon Eclipse Ts2R fluorescence microscope or Nikon Ti2E fluorescence microscope. For higher magnification confocal images, the Olympus FV3000 confocal microscope was used. A Nikon Ti2E fluorescence microscope was used for live imaging, with three random positions per well selected. ImageJ, Adobe Photoshop CS6 and Illustrator CS6 were used to process images.

Mitochondrial morphology was categorized as ‘fragmented’ when most of the cell’s mitochondria appeared short and spherical, ‘hyperfused’ when most mitochondria were highly elongated with fewer than ten free ends and ‘intermediate’ when most mitochondria displayed a tubular morphology that was neither extensively connected nor spherical, referencing previous studies^31^. At least 60 cells per condition were analyzed. For peroxisomal area analysis, max projection images were processed by manual thresholding. The peroxisomal area was obtained using the ‘Analyze particles’ plugin in Fiji with a minimum area of 0.1 μm^2^. At least 20 cells per condition were analyzed.

Quantification of CC intensity (CC bundle index) was calculated by dividing the average GFP intensity of the CC by the number of electroporated cells. For ipsilateral axon branching and layer 2/3 intensity analysis, the region of interest was designated within the electroporated area, with minimal nonmigrating cells. The contralateral and ipsilateral regions of interest were set to be symmetrical about the midline. Fluorescence intensity was quantified using the Fiji Plot Profile tool, and values were normalized by subtracting the fluorescence intensity recorded in the darkest region of the brain.

### Statistical and data analysis

The study was conducted without predetermined sample size calculations or statistical power analyses. Data collection proceeded without predefined stopping criteria. All collected data were included in the analysis. Experiments were performed at least twice with consistent results and included a minimum of three biological replicates unless otherwise noted. All genotype, treatment and cortical bin quantification were conducted by researchers blinded to conditions.

All statistical analyses were performed using R 4.2.2, GraphPad Prism 10 (Graphpad Software) and MS Excel (Microsoft). When data did not follow a normal distribution, the Kruskal–Walis test was used to compare multiple means. When data followed a normal distribution but did not meet homogeneity of variance, Brown–Forsythe and Welch’s analysis of variance (ANOVA) was applied. Otherwise, one-way or two-way ANOVA was used to calculate *P* values. A Mann–Whitney *U* test was used to compare the means of the two groups if the data did not follow a normal distribution. Otherwise, Student’s *t* test and Welch’s *t* test were used depending on whether the data met homogeneity of variance. We used Tukey’s, Dunn’s, Bonferroni’s or Dunnett’s method for *P* value adjustment for appropriate multiple comparisons. Data are presented as the mean ± s.e.m. In all cases, significance was accepted for *P* values <0.05, and *P* values <0.05 (^*^), <0.01 (^**^), <0.001 (^***^) or <0.0001 (^****^) were indicated on the data plots. Biological replicates and statistical tests used for comparisons are indicated in the figure legends.

## Results

### Novel *DNM1L* mutations in two DAE cases

**Fig. 1:**
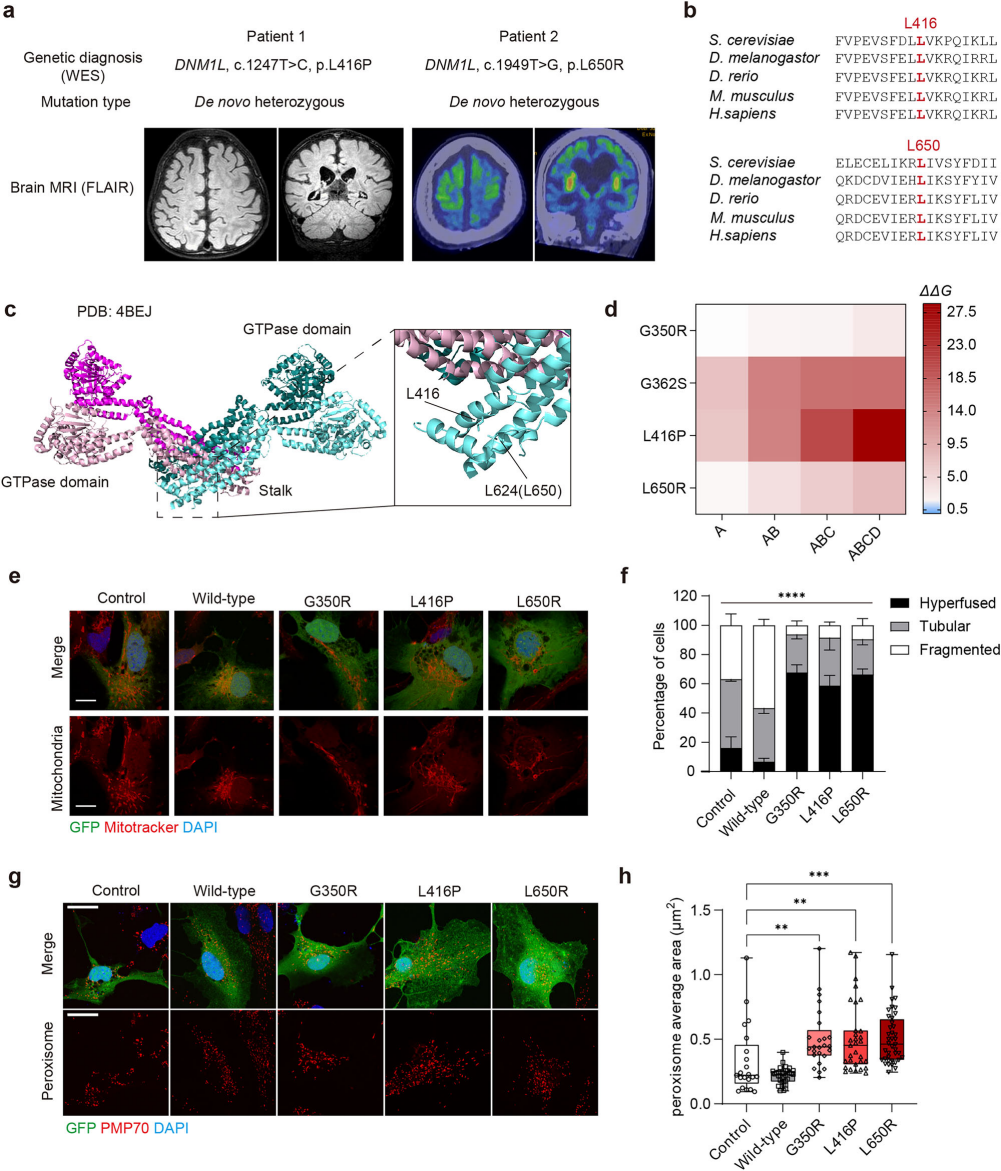
Novel DNM1L mutations identified in patients with status epileptic encephalopathy. **a** Mutation information and representative MRI images of two patients. The T2 axial view of patient 1 was taken at the age of 3 years and 9 months. The FLAIR axial view of patient 2 was taken at the age of 1 year and 10 months, with increased signal intensity indicating atrophic change. **b** Cross-species conservation of protein sequences near the mutation residue. **c** The structure of the DRP1 tetramer (PDB, 4BEJ) shown as a ribbon diagram, with each protomer colored differently. Mutated residues are highlighted as sticks and labeled in a zoom-in box. L650 (UniProt ID, O00429-1) corresponds to L624 in DNM1L isoform 2 (UniProt ID, O00429-3) in the displayed structure. **d** Perturbation modeling of previously reported (G350R and G362S) and novel (L416P and L650R) DNM1L mutants using FoldX. A, B, C and D represent chain IDs in the tetramer structure, while AB, ABC and ABCD correspond to the dimer, trimer and tetramer, respectively. **e** Representative images of mitochondrial morphology in human NPCs transfected with *DNM1L* variants. Red, Mitotracker CMXRos; green, GFP; blue, DAPI. Scale bar, 10 μm. **f** Quantification of mitochondrial morphology in human NPCs transfected with *DNM1L* variants. *n* = 3 with at least 60 cells in each condition analyzed for an independent experiment. **g** Representative images of peroxisomal morphology in human NPCs transfected with *DNM1L* variants. Red, PMP70; green, GFP; blue, DAPI. Scale bar, 20 μm. **h** Quantification of peroxisomal morphology in human NPCs transfected with *DNM1L* variants. Control, *n* = 21; wild type, *n* = 34; G350R, *n* = 25; L416P, *n* = 31; L650R, *n* = 40. Bar graphs indicate mean ± s.e.m. Statistical significance is determined by two-way ANOVA with Tukey’s post hoc test for **e** and the Kruskal–Wallis test with Dunn’s post hoc test for **h**. ^**^P < 0.01; ^***^P < 0.001; ^****^P < 0.0001.

We clinically identified novel de novo heterozygous missense mutations (c.1247T>C, p.L416P and c.1949T>G, p.L650R, respectively) of *DNM1L* in two individuals (patient 1 and patient 2) from unrelated families presenting with facial dysmorphism, developmental regression and developmental epileptic encephalopathies. Both patients exhibited diffuse atrophic changes in brain MRI and markedly abnormal electroencephalogram (EEG) backgrounds (Fig. 1a and Supplementary Fig. 1a–d). The T2 axial MRI view of patient 1 demonstrated diffuse brain atrophy in the cortex, with positron emission tomography brain scan showing decreased ^18^F-fluorodeoxyglucose uptake in the right sensorimotor area, indicating possible neuronal loss or dysfunction in this region. An EEG of patient 1 taken at the age of 3 years and 8 months showed markedly abnormal background with abundant, nearly continuous generalized slow sharp and wave discharges frequently interrupted by electrodecrement. The fluid-attenuated inversion recovery (FLAIR) axial MRI view of patient 2 also revealed increased signal intensities in bilateral middle and inferior frontal areas, CC, anterior cingulate areas, and hippocampi, showing a sign of diffuse brain atrophy. An EEG of patient 2 taken at the age of 4 years and 5 months showed slow and disorganized background rhythms and the presence of nearly continuous, repetitive, synchronous slow generalized spike-and-wave discharges, intermixed with brief electrodecrement.

In silico analysis using variant effect prediction tools classified both variants to be pathogenic (Supplementary Table 1). The mutated residues were highly conserved across species and located near the self-assembly interface at the end of the stalk domain, which consists of the middle and GTPase effector domains (Fig. 1b,c). DRP1 forms the higher-order oligomeric ring at the mitochondria–endoplasmic reticulum contact sites and constricts the membrane through GTP hydrolysis^32–35^. Perturbation modeling by FoldX predicted that L416P and L650R destabilize the DRP1 tetramer, as indicated by an increase in ΔΔ*G* with the number of mutations introduced^36^ (Fig. 1d). Thus, we hypothesized that the two novel mutants function as dominant negative as seen in the majority of *DNM1L* variants (Supplementary Fig. 1e). Indeed, in human NPCs, expression of *DNM1L*^*L416P*^ or *DNM1L*^*L650R*^ resulted in a hyperfused mitochondrial network and elongated peroxisomes, comparable to the expression of G350R, a previously reported dominant negative mutation, whereas overexpression of the wild type *DNM1L* increased the fragmented mitochondrial and peroxisomal morphology compared with the vector control^3^ (Fig. 1e–h).

Both patients developed severe drug-resistant epilepsy over time rather than at birth. Patient 1 developed focal hemiclonic status epilepticus beginning at 16 months of age with repetitive myoclonic jerks. Patient 2 initially presented with vacant staring and head drop at 6 months, progressing to status epilepticus after a 4-year seizure-free period. Both patients exhibited developmental delay before seizure onset, followed by regression with severe spasticity after seizure onset. However, the onset of the symptom deterioration was unclear. Moreover, despite the administrations with anticonvulsants, vitamin cocktails and ketogenic diets on the basis of the genetic diagnosis of a mitochondrial disorder, the seizures and developmental regression could not be halted, raising the urgent necessity of research into the timing, reversibility and treatability of DAE neuropathology.

### Postnatal neuropathology in DAE mouse model

**Fig. 2:**
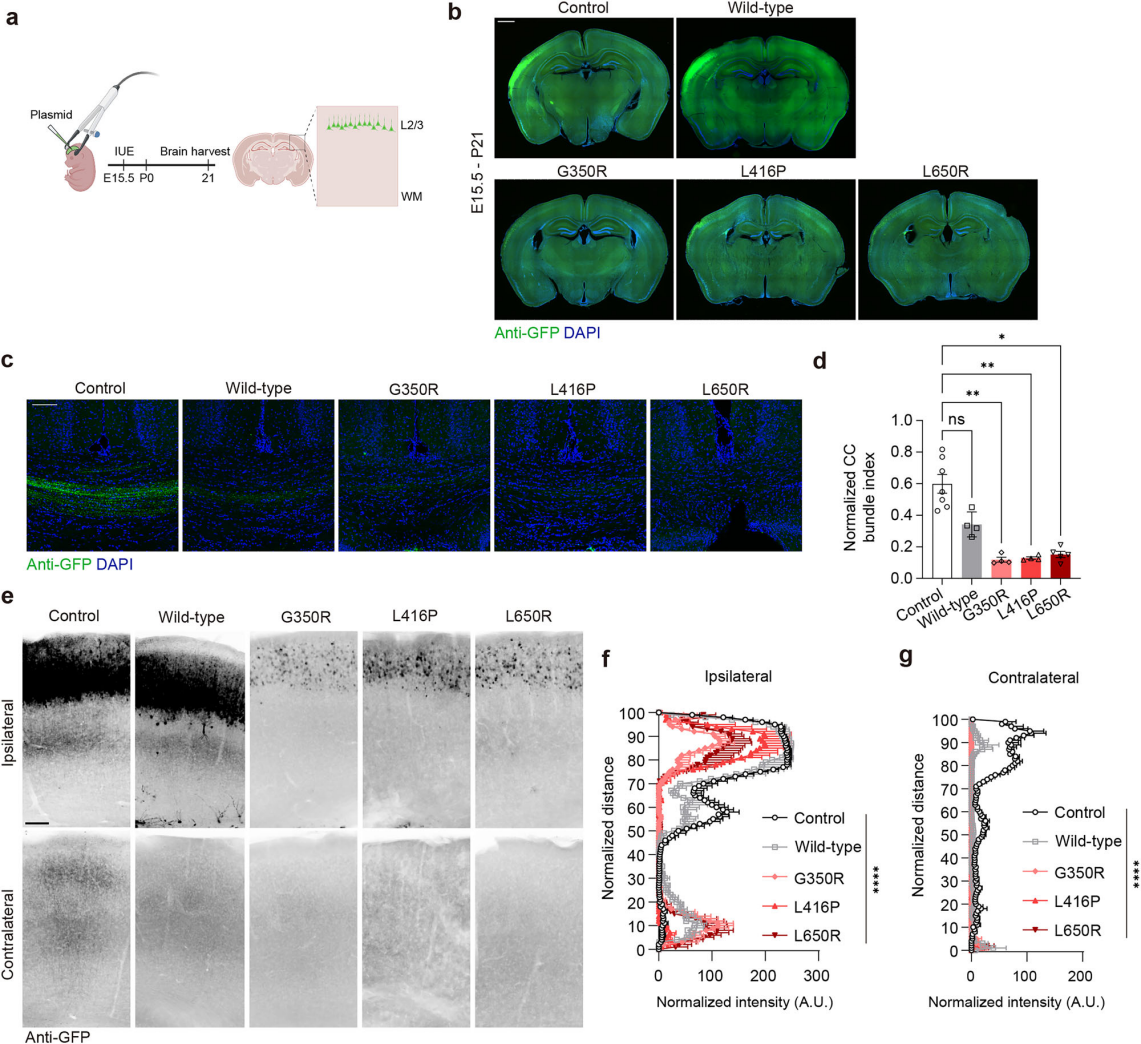
Modeling DAE in the developing mouse brain. **a** Scheme of DAE mouse model generation via IUE. **b** Representative images of brain slices electroporated with each variant and stained with anti-GFP. Green, GFP; blue, DAPI. Scale bar, 1 mm. **c**, **d** Representative confocal *z*-stack images and quantification of 35-µm sections of a P21 mouse brain CC immunostained with anti-GFP. Green, GFP; blue, DAPI. Scale bar, 100 μm. Control, *n* = 7; wild type, *n* = 4; G350R, *n* = 4; L416P, *n* = 4; L650R, *n* = 5. **e** Representative images of P21 ipsilateral and contralateral cortical plates electroporated with each variant. Slices were immunostained with anti-GFP. Black, GFP. Scale bar, 200 μm. **f** Quantification of P21 ipsilateral cortical plates electroporated with each variant. Control, *n* = 7; wild type, *n* = 4; G350R, *n* = 4; L416P, *n* = 3; L650R, *n* = 5. **g** Quantification of P21 contralateral cortical plates electroporated with each variant. Control, *n* = 7; wild type, *n* = 4; G350R, *n* = 4; L416P, *n* = 3; L650R, *n* = 5. Bar plot indicates mean ± s.e.m. Statistical significance is determined by the Kruskal–Wallis test with Dunn’s post hoc test for **d** and two-way ANOVA with Bonferroni’s post hoc test for **f** and **g**. ^*^P < 0.05; ^**^P < 0.01; ^****^P < 0.0001. Panel **a** created with BioRender.com.

To understand the neuropathological impact of *DNM1L* variants across developmental and postnatal stages, we developed a mouse model that introduced the vectors expressing GFP and human *DNM1L* variants into the developing mouse cortex via in utero electroporation (IUE) (Fig. 2a). IUE was timed at the E15.5 when the genesis of callosal projection neurons peaks^37^. The IUE model provides a practical approach to test multiple dominant negative variants while minimizing the risk of embryonic lethality by restricting the expression of these variants to a subset of neurons.

**Fig. 3:**
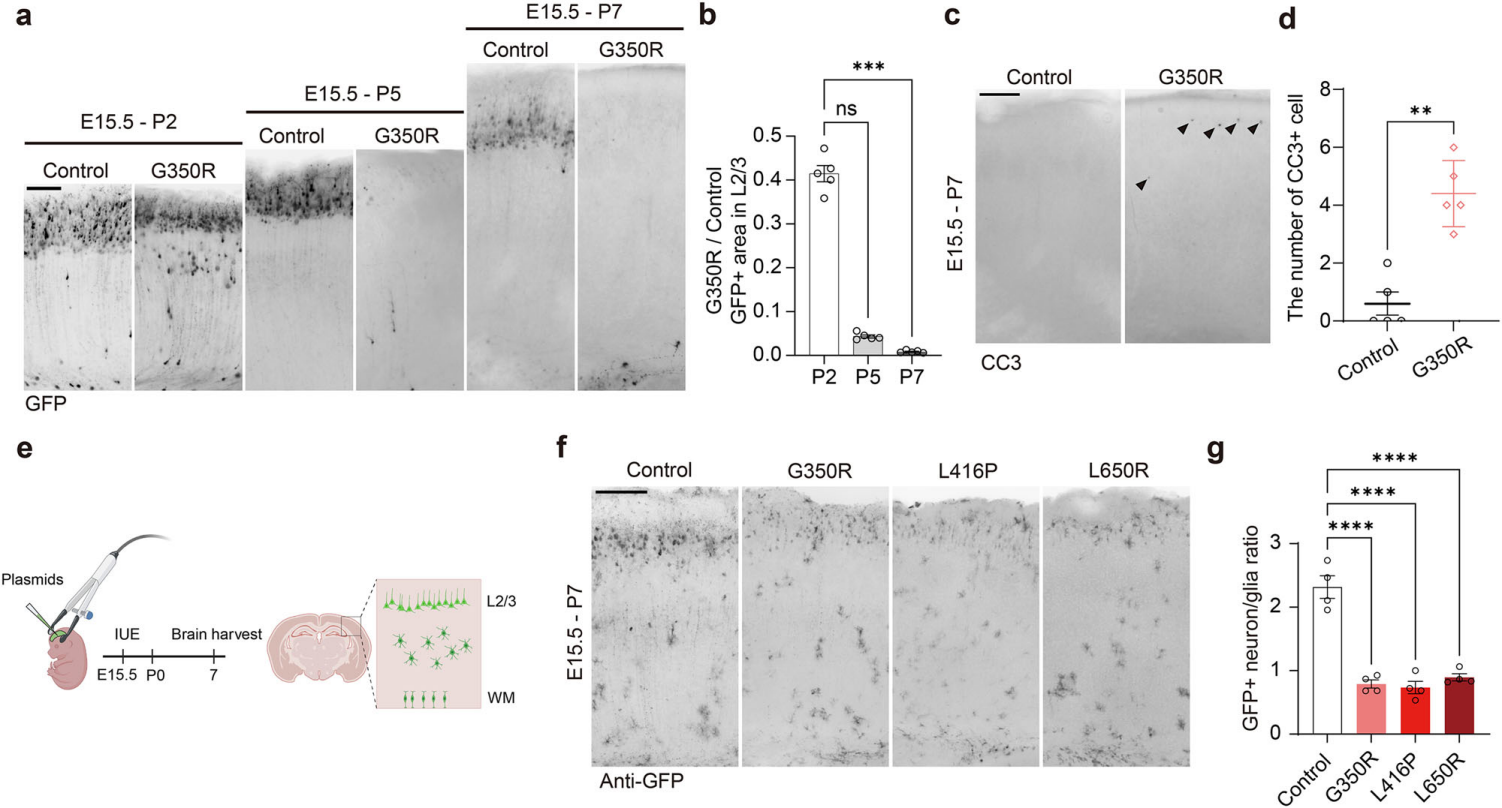
Postnatal neuronal loss caused by DNM1L variants in the mouse model. **a**, **b**, Representative images and quantification of GFP signal in postnatal brain slices electroporated with control vector or *DNM1L*^*G350R*^. IUE timelines are indicated above. Black, GFP. Scale bar, 100 μm. **b** Quantification of GFP^+^ area ratio (G350R/control) in postnatal brain slices. *n* = 5. **c**, **d** Representative images and quantification of P7 mouse brain sections immunostained for CC3. Black arrows indicate positive cells. Black, CC3. Scale bar, 200 μm. *n* = 5. **e** IUE scheme for piggyBac system to label neurons and glia. **f**, **g** Representative images and quantification of P7 mouse brain sections electroporated with piggyBac system and immunostained with anti-GFP. Black, GFP. Scale bar, 200 μm. *n* = 4. Bar plot indicates mean ± s.e.m. Statistical significance is determined by the Kruskal–Wallis test with Dunn’s post hoc test for **b**, the Mann–Whitney test for **d** and one-way ANOVA with Dunnett’s post hoc test for **g**. ^**^P < 0.01; ^***^P < 0.001; ^****^P < 0.0001; n.s., not significant. Panel **e** created with BioRender.com.

On P21, we observed that mouse brains expressing *DNM1L*^*L416P*^ *or DNM1L*^*L650R*^ showed a reduction in cell number, axon branching and CC dysgenesis compared with controls (Fig. 2b–g). These defects were also presented in *DNML1L*^*G350R*^-expressing brains but not in wild type *DNM1L*-expressing brains, indicating the neuropathological impact resulted from dominant-negative mutations rather than simple overexpression. We then investigated when the observed pathologies were exacerbated, as the diminished neuron number in mouse brains expressing *DNM1L* variants may be explained by reduced proliferation and/or increased cell death. In the prenatal stages, the expression of *DNM1L* mutations did not significantly affect apoptosis or proliferation (Supplementary Fig. 2a–e). The differentiation defect, indicated by the percentage of Satb2-positive cells among GFP-positive electroporated cells, was not observed at E18.5 (Supplementary Fig. 2f,g). Although the expression of *DNM1L* variants resulted in varying degrees of migration defects, this does not fully account for the observed pathologies at P21 (Supplementary Fig. 2h,i). At postnatal stages, however, a significant loss of GFP-positive cells was observed in mice brains expressing *DNM1L*^*G350R*^ (Fig. 3a,b). Increased apoptosis was also detected in *DNM1L*^*G350R*^-expressing brains at P7, suggesting a progressive neuronal loss during the postnatal stage (Fig. 3c,d). Of note, the expression of *DNM1L*^*E2A*^, a restricted-dominant negative found in patients with milder clinical presentation, did not cause neuronal loss to a similar degree^8^ (Supplementary Fig. 3).

Given that our models restrict *DNM1L* variant expression to upper-layer neurons, we used the piggyBac vector system, which labels progeny of electroporated radial glial cells at E15.5, allowing us to examine the outcome of *DNM1L* variant expression in late-born glial cells as well^38^ (Fig. 3e). In brains that expressed control vectors, cells with glial morphology were observed across the cortical plate in addition to neurons in the upper cortical layer. In brains expressing *DNM1L* variants, the neuronal population in the upper cortical layer was diminished, while cells with glial morphology were similar in number, as indicated by a decreased neuron-to-glia ratio in the cortical plate at P7 compared with the control (Fig. 3f,g). Taken together, our model presented postnatal neuronal loss as a key histological pathology caused by *DNM1L* variants, which may offer a possible explanation for the clinical presentations observed in patients, such as cerebral atrophy and CC thinning.

### Neurodevelopmental stage-specific transcriptomic perturbations induced by *DNM1L* dysfunction

Next, to further profile the molecular pathology caused by *DNM1L* dysfunction during the differentiation of NPCs into neurons, we established an inducible *DNM1L* KO (DNM1L^iKO^) model using H9 human ES cells. We selected human ES cells for efficient clonal selection and expansion, as well as robust derivation of NPCs recapitulating early neurodevelopmental stages. To bypass lethality caused by complete loss of *DNM1L*, we introduced wild type *DNM1L* using a Tet-off system followed by targeted KO of endogenous *DNM1L* allele by CRISPR–Cas9 before NPC differentiation (Supplementary Fig. 4a). Upon DOX treatment, NPCs derived from the clone containing compound heterozygous mutations in endogenous *DNM1L* loci showed reduced DRP1 protein levels compared with the control (Supplementary Fig. 4b,c). Indeed, DOX treatment resulted in hyperfused mitochondrial networks and elongated peroxisomes similar to *DNM1L* KO phenotypes reported in other cell types^1,9,13,16,17^ (Supplementary Fig. 4d–f). The KO phenotypes were rescued by wild type *DNM1L* expression but not by *DNM1L*^*G350R*^, *DNM1L*^*L416P*^ or *DNM1L*^*L650R*^, confirming our results in Fig. 1 (Supplementary Fig. 4d–f).

**Fig. 4:**
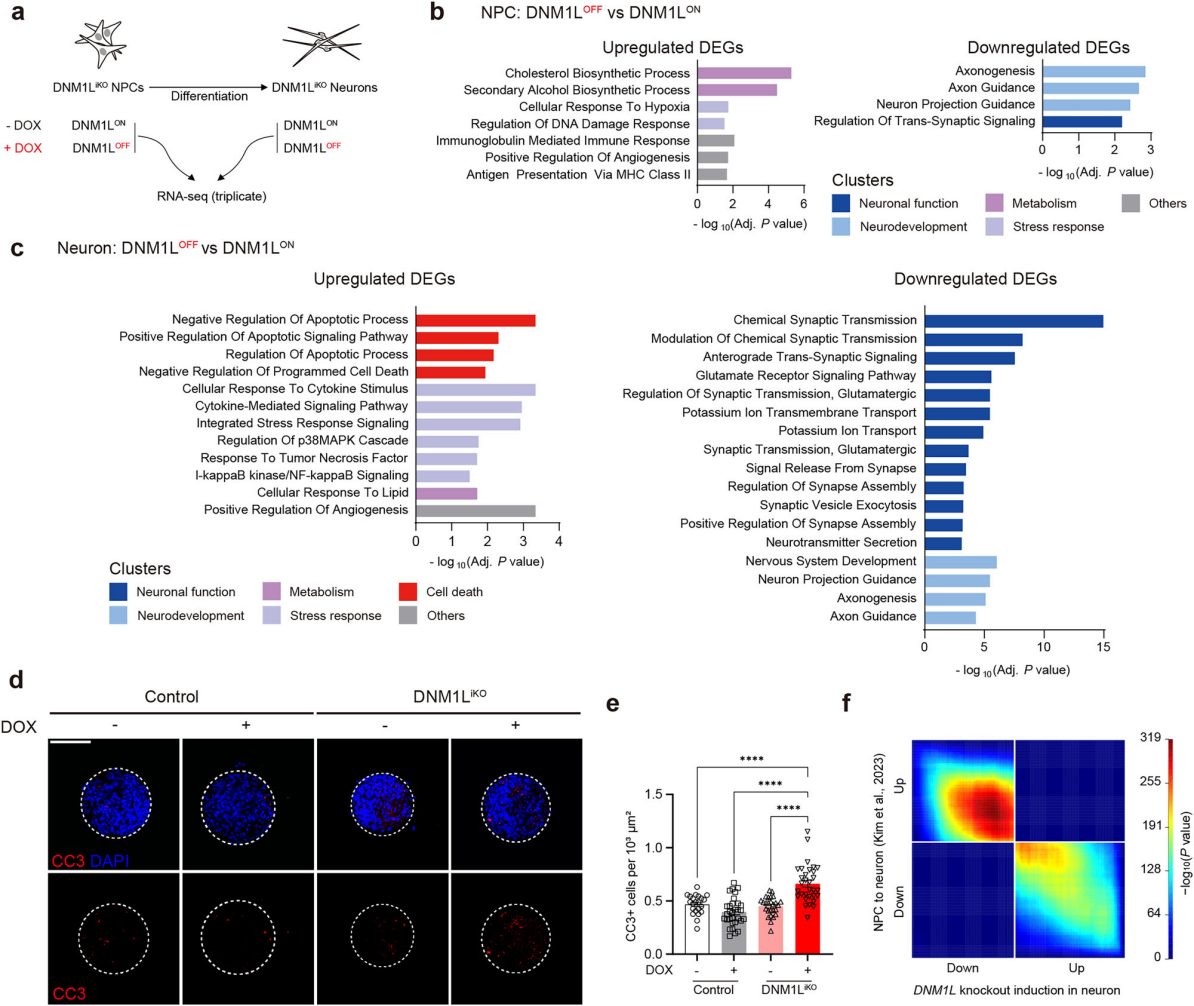
Transcriptomic profiling in the DNM1L^iKO^ NPCs and neurons. **a** Scheme of RNA sequencing sampling from DNM1L^iKO^ NPCs and differentiated neurons. Cells were treated with DMSO (DNM1L^ON^) or DOX (DNM1L^OFF^) during culture. **b**, **c** Functional enrichment analysis of upregulated and downregulated DEGs between DNM1L^OFF^ and DNM1L^ON^ conditions in NPCs (**b**) and neurons (**c**). The bar color indicates the cluster categories of the GO term. **d** Representative images of neurospheres immunostained with CC3. Red, CC3; blue, DAPI. Scale bar, 50 μm. **e** Quantification of neurospheres immunostained with CC3. Control Dox−, *n* = 22; control Dox+, *n* = 31; DNM1L^iKO^ Dox−, *n* = 26; DNM1L^iKO^ Dox+, *n* = 31. **f** RRHO analysis. The overlap of gene expression alterations by *DNM1*L dysfunction (*x* axis, DNM1L^OFF^ versus DNM1L^ON^) and by neuronal differentiation (*y* axis) was compared. Genes were ordered on the basis of their Wald test statistics (log_2_ fold change divided by the standard error of the log_2_ fold change). Each point on the plot indicates the significance level of the overlap between the two ranked gene lists. Bar graphs indicate mean ± s.e.m. Statistical significance is determined by Brown–Forsythe and Welch ANOVA with Dunnett’s T3 post hoc test for **e**. ^****^P < 0.0001.

We then examined the transcriptomic changes in DNM1L^iKO^ at two key developmental stages (Fig. 4a). The effect of DOX was filtered out by comparing transcriptional changes in control NPCs derived from H9 parental cells with or without DOX treatment. Control cells were referred to as DNM1L^ON^, while DOX-treated samples were referred to as DNM1L^OFF^. GO enrichment analysis of DEGs between DNM1L^OFF^ and DNM1L^ON^ at NPC and neuronal stages revealed that they could be classified into several major categories: (1) neuronal function, (2) development, (3) metabolism, (4) stress response, (5) cell death and others. At NPC stages, GO analysis revealed that 637 upregulated DEGs were significantly enriched in metabolism and stress response, including cholesterol biosynthesis (*MSMO1*, *CYP51A1* and *HSD17B1*) and cellular response to hypoxia (*VEGFA*, *PLK3* and *PMAIP1*), respectively, while 868 downregulated DEGs were overrepresented in neurodevelopment such as axonogenesis (*SLITRK5*, *EPHB1* and *SEMA5B*) (Fig. 4b). At neuronal stages, GO analysis of 382 upregulated DEGs highlighted enrichment in cell death and stress response, including apoptosis regulation (*NUPR1*, *BIRC3*, *DDIT* and *BID*) (Fig. 4b). Indeed, the apoptosis level was higher in DOX-treated DNM1L^iKO^ neurospheres (Fig. 4d,e). Stress response-related DEGs such as tumor necrosis factor and NFκB signaling genes (*SPHK1*, *NFKBIA* and *RELB*), which have also been known for their possible roles in mediating epileptogenesis and neuronal death, were also enriched^39–41^. By contrast, 729 downregulated DEGs were enriched in neuronal functions related to synaptic transmission and ion channel activity (*GABRB2*, *GRIK2*, *KCND2* and *CACNA1B*) (Fig. 4c). We analyzed transcriptome data with RRHO to test if *DNM1L* dysregulation delayed neuronal maturation. While 50.3% of upregulated genes in DOX-treated DNM1L^iKO^ neurons overlapped with downregulated genes during neuronal differentiation, 64.4% of downregulated genes in DOX-treated DNM1L^iKO^ neurons showed more significant overlap with genes upregulated during neuronal differentiation, suggesting delayed neuronal maturation^25,42^ (Fig. 4f).

In addition, upon H_2_O_2_ stress, the percentage of late apoptotic and necrotic neuronal populations increased in DOX-treated DMN1L^iKO^ NPCs compared with controls (Supplementary Fig. 5a–d). Enriched GO terms related to stress response may explain the elevated susceptibility to stress-induced cell death caused by *DNM1L* dysfunction (Fig. 4b). Taken together, our results revealed transcriptional signatures underlying neuronal loss affected by *DNM1L* dysfunction during the key stages of human neuronal development. Moreover, transcriptional changes related to cell death were more pronounced in differentiated neurons, aligning with postnatal neuropathology in the mouse model.

### Transcriptional reversibility via chemogenetic rescue in differentiated neurons

**Fig. 5:**
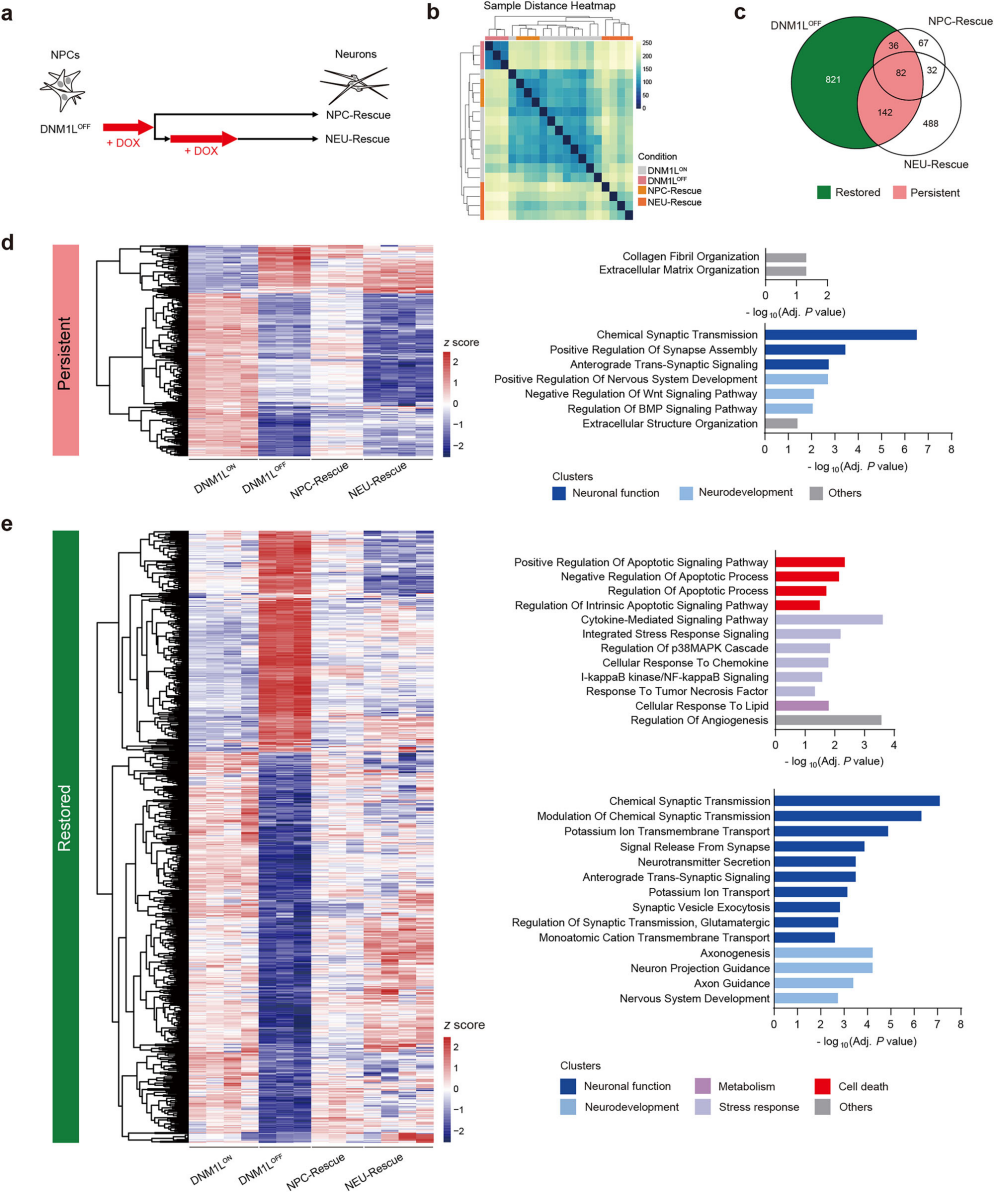
Transcriptomic reversibility in DNM1L^iKO^ neurons by chemogenetic recovery of DNM1L. **a** Schematic diagram of rescue conditions. Red arrows indicate DOX treatment during culture. **b** Heat map of sample-to-sample distances based on variance-stabilized transformation of read count data for total gene expression. The clustering of RNA sequencing samples illustrates the genetic relationships, as indicated by the intensities of the square colors. **c** Overlapped DEGs between the indicated comparison groups are shown as a Venn diagram. DEGs of each group were identified by comparison with the DNM1L^ON^ group. Restored and persistent DEGs in DNM1L^OFF^ condition are filled with green and pink, respectively. **d**, **e** Heat maps (left) and functional enrichment analysis (right) of persistent (**d**) and restored (**e**) DEGs. The color gradient in the heat map represents the *Z* score. Functional enrichment of upregulated and downregulated genes was shown in separate bar plots. The bar color indicates the cluster categories of the GO term.

To test transcriptomic reversibility during the key stages of human neuronal development, we chemogenetically restored the *DNM1L* expression by DOX withdrawal during or after early neuronal differentiation (NPC-Rescue or NEU-Rescue, respectively) (Fig. 5a). The recovery of *DNM1L* expression at both the mRNA and protein levels, as well as the morphology of peroxisomes and mitochondria, after DOX withdrawal demonstrates the robustness of the methodology (Supplementary Fig. 6a–d). Principal component analysis of batch-corrected RNA sequencing data revealed the clustering of control sets from different batches, allowing reliable further meta-analysis (Supplementary Fig. 6e).

We found that both NPC-Rescue and NEU-Rescue clustered closer to DNM1L^ON^ controls on the sample distance heat map than DNM1L^OFF^ (Fig. 5b). Only 10.9% and 20.7% of DEGs seen in DNM1L^OFF^ were found in NPC-Rescue and NEU-Rescue, respectively, indicating that the majority of transcriptional changes can be rescued during or after early neuronal differentiation (Fig. 5c). We classified the DEGs by *DNM1L* dysfunction into two categories—‘restored’ and ‘persistent’—on the basis of their expression changes after the recovery of *DNM1L* expression at two different stages. We identified that 24.1% (260/1081) of DEGs remained persistent (Fig. 5d). Among these, 36 were specific to NPC-Rescue, 142 were specific to NEU-Rescue and 82 remained persistent regardless of the stage at which *DNM1L* expression was recovered (Fig. 5c). Given that NPC-Rescue showed fewer persistent DEGs, we assessed potential compensation through cell division in NPC-Rescue with time course analysis at the cellular and molecular levels. No rescue-specific proliferation was detected during early neuronal induction (Supplementary Fig. 7). However, compensation at the molecular level, such as *CDKN1A*, may indicate partial restoration of cell cycle regulation (Supplementary Fig. 8).

We also identified that 75.9% (821/1081) of DEGs were restored regardless of *DNM1L* rescue stages (Fig. 5e). GO analysis of restored DEGs showed enrichment in cell death, stress response, neuronal function and neurodevelopment among six categories altered by *DNM1L* dysfunction, although persistent DEGs were also enriched in neuronal function and neurodevelopment (Fig. 5e). SynGO analysis of persistent DEGs revealed enrichment into synaptic organization and signaling. By contrast, restored DEGs showed an overrepresentation of trans-synaptic signaling and pre- and postsynaptic membrane potential regulation in SynGO analysis (Supplementary Fig. 9a).

The potential transcription factors of persistent and restored DEGs were inferred using ChEA3^43^ (Supplementary Fig. 9b). The top-ranked transcription factors for persistent DEGs included developmental homeobox genes (for example, *LHX1*, *LHX5* and *DBX2*). However, the top-ranked upstream regulators of restored DEGs were mainly associated with neuronal differentiation (for example, *MYT1, SCRT1* and *MYT1L*) and early responses in neurons (for example, *EGR4* and *NPAS4*).

These data demonstrate that the majority of transcriptional changes caused by *DNM1L* dysfunction, including those associated with cell death, are restorable in differentiated neurons, which may pose treatment potential in the postnatal time window.

### Amelioration of neuronal loss by perinatal mitochondrial biogenesis enhancement

**Fig. 6:**
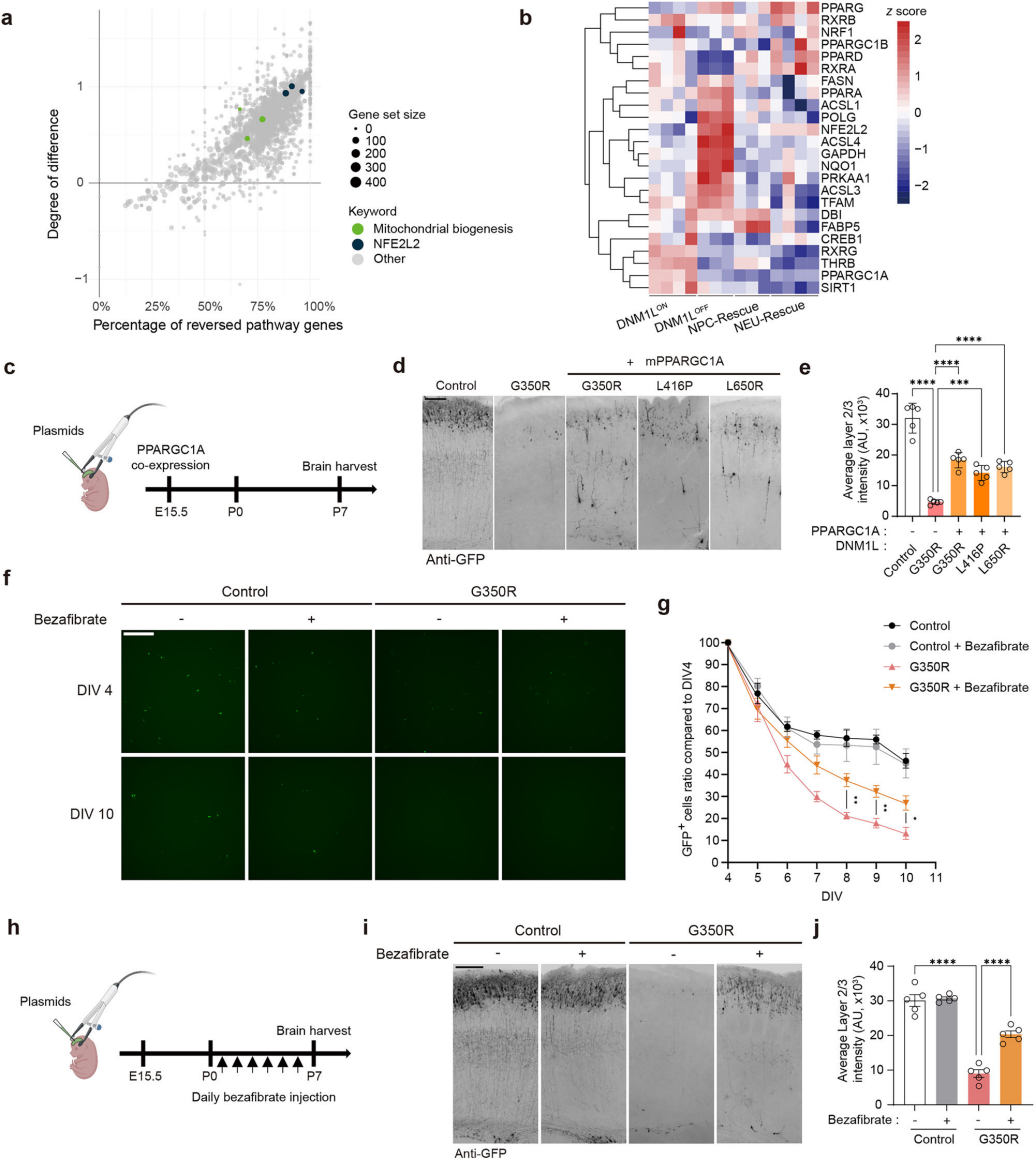
Prevention of neuronal loss by perinatal mitochondrial biogenesis enhancement. **a** Scatter plot presenting reversibility of Reactome pathways, color-coded by keyword and sized by gene set size. The *x* axis is the percentage of reversible genes among the Reactome pathway gene sets, and the *y* axis is the difference in the extent of change caused by *DNM1L* dysfunction and under rescue conditions compared with control, expressed as a *Z* score. **b** Heat map of PPAR–PGC1α signaling pathway genes in different conditions. The color gradient represents *Z* scores. **c** Scheme of mimicking reversible effects by overexpressing the mitochondrial biogenesis master regulator *PPARGC1A*. **d**, **e** Representative images and quantification of P7 *PPARGC1A*-coexpressing mouse brain sections immunostained with anti-GFP. Black, GFP. Scale bar, 100 μm. *n* = 5. **f**, **g** Representative images and quantification of mouse primary cortical neurons electroporated with control or *DNM1L*^*G350R*^, treated with DMSO vehicle or bezafibrate. Green, GFP. Scale bar, 50 μm. The viability of mouse primary cortical neurons was compared with the starting point DIV 4. *n* = 6 from two independent experiments for all conditions. **h** Scheme of bezafibrate administration during the perinatal period. **i**, **j** Representative images of P7 mouse brain sections treated with DMSO vehicle or bezafibrate and immunostained with anti-GFP. Black, GFP. Scale bar, 100 μm. *n* = 5. Bar graphs indicate mean ± s.e.m. Statistical significance is determined by one-way ANOVA with Tukey’s post hoc test for **e** and **j** and two-way ANOVA with Bonferroni’s post hoc test for **g**. ^*^P < 0.05, ^**^P < 0.01, ^***^P < 0.001, ^****^P < 0.0001. Panels **c** and **h** created with BioRender.com.

To identify targetable nodes that may alleviate the major neuropathology caused by *DNM1L* mutations identified in our models, we analyzed Reactome pathways on the basis of two variables^44^. The first variable is the difference (*Z* score) in the extent of change caused by *DNM1L* dysfunction and under rescue conditions compared with control, while the second variable is the percentage of reversible genes among the Reactome pathway gene sets (Fig. 6a). Antioxidant *NFE2L2* signaling was among the pathways exhibiting the highest reversibility: the rescue conditions were closer to the control than the *DNM1L* dysfunction conditions, with about 92% of genes in the pathway gene set being reversible. By contrast, histone modification and senescence showed the opposite (Supplementary Fig. 9c). Consistent with the GO analysis (Fig. 5e), the apoptosis and potassium channel-related pathways also showed high reversibility (Supplementary Fig. 9d).

Among these, we focused on the mitochondrial biogenesis pathway to rescue neuronal loss^45,46^ (Fig. 6a). Among mitochondrial biogenesis-related PPAR–PGC1α pathway genes, 62.5% were restorable upon chemogenetic rescue (Fig. 6b). We therefore reasoned that targeting the upstream regulator *PPARGC1A* might mimic the reversible effects. To test the hypothesis, we co-expressed the mouse *PPARGC1A* gene with *DNM1L* variants in the developing mouse brain (Fig. 6c). While the expression of *DNM1L*^*G350R*^ caused a reduction in GFP-positive neurons, co-expression of *PPARGC1A* significantly rescued neuronal loss (Fig. 6d,e). Moreover, neuronal loss caused by other *DNM1L* variants was also rescued by co-expression of *PPARGC1A*.

We next investigated whether the neuropathies caused by *DNM1L* dysfunction could be treated with small molecules that mimic the co-expression of *PPARGC1A*. Bezafibrate is one of such small molecules that activate the PPAR–PGC1α signaling pathway by enhancing the *PPARGC1A* level^45–47^. We thus examined the effect of bezafibrate on the mouse primary cortical neurons expressing *DNM1L*^G350R^. The number of *DNM1L*^G350R^-expressing mouse primary cortical neurons declined rapidly from DIV 4 to 10, which was rescued by bezafibrate (Fig. 6f,g). Finally, we assessed the treatment feasibility in the vulnerable postnatal period. To maximize the therapeutic effect, daily intraperitoneal bezafibrate injections were administered to mice expressing control or *DNM1L*^G350R^ variant from P0 to P6 (Fig. 6h). *DNM1L*^G350R^-electroporated mice injected with bezafibrate showed significantly increased survival of GFP-positive cells in contrast to those injected with vehicle (Fig. 6i,j).

These results show that genetic or pharmacological activation of the mitochondrial biogenesis pathway could prevent the neuropathological hallmark caused by the *DNM1L* dysfunction at the histological level in the perinatal stage, suggesting the potential postnatal feasibility of clinical intervention in treating *DNM1L*-related neurodevelopmental disorders.

## Discussion

The majority of patients with DAE present with brain MRI abnormalities, such as cerebral atrophy and CC thinning, which have been replicated in our mouse model (Fig. 2). The alignment of postnatal neuronal loss in the mouse model with the early onset of symptoms in patients underscores the significance of postnatal defects. Although controversial, previous studies have suggested that neuronal loss may precede the onset of seizures and contribute to certain forms of epilepsy^48,49^. However, the precise progression of symptoms and the relationships between different manifestations, such as cerebral atrophy and seizures, remain elusive owing to limited longitudinal clinical data. This necessitates long-term comprehensive monitoring of patients with DAE to refine clinical interventions. Moreover, the heterogeneity of neurological outcomes among patients with DAE poses challenges for translational clinical practices. Emerging genotype–phenotype correlations in *DNM1L*-related disorders suggest that mutations in the stalk domain are associated with more severe symptoms such as psychomotor retardation and epilepsy^12,13,50^. Our mouse model partially supports this observation, as *DNM1L*^G350R^, *DNM1L*^L416P^ and *DNM1L*^L650R^ mutations led to postnatal neuropathology, whereas the *DNM1L*^E2A^ mutation, which is associated with relatively milder dominant optic atrophy, did not^8^ (Fig. 2 and Supplementary Fig. 3). The three variants examined in this study all map to the stalk domain that is critical for DRP1 assembly. In silico analysis predicted oligomer destabilization for all three variants, consistent with the observed mitochondrial hyperfusion and peroxisomal elongation in human NPCs and with postnatal neuronal loss in the mouse IUE model, suggesting a converging impact on neuronal vulnerability. Clinically, similar to our patient cases, *DNM1L*^*G350R*^ has been associated with severe progressive cerebral volume loss and status epilepticus^3^. These findings suggest that stalk-domain mutations correlate with severe presentations and provide a rationale for tailoring therapeutic strategies to mutation class and domain context.

Our results propose two possible explanations for the neuronal loss caused by *DNM1L* mutations, which predominantly occur during the first postnatal week. The first possibility is the stress response due to mitochondrial dysfunction. Our transcriptional profiling data showed the enrichment of upregulated DEGs by *DNM1L* dysfunction in apoptosis and stress response, indicating the heightened vulnerability of differentiating neurons to *DNM1L* dysfunction (Fig. 4c–e). The second possibility could be the elimination of neurons with synaptic dysfunction during the perinatal period, a critical window for refining neural circuits by removing neurons exhibiting abnormal activity^51–53^. Beyond known intrinsic regulators of programmed cell death, such as BCL-2 and BAX, it has been postulated that aberrant neural activity may be a possible determinant in neuronal survival during the early postnatal neurodevelopment^51,52,54^. *DNM1L*-dysfunctioned neurons are therefore probably prime targets for removal, given that they have been shown to exhibit axonal growth and synapse formation defects, resulting in abnormal neural activity^18,55,56^.

Our findings demonstrated the feasibility of treating DAE at histological and transcriptional levels using in vivo and in vitro models. We observed transcriptional reversibility upon *DNM1L* expression recovery, with over three-quarters of DEGs restorable in differentiated neurons, suggesting high plasticity at the transcriptional level before irreversible neuronal loss. Restoration of mitochondrial dynamics balance has been proposed as a potential therapeutic intervention for related diseases, where reintroduction of *Mfn2* in cerebellar conditional KO models prevented neurodegeneration^57^. Moreover, our results demonstrated that enhancing mitochondrial biogenesis significantly rescued neuronal loss in the mouse model, even during the postnatal stage. Mitochondrial biogenesis has been proposed as a therapeutic target in several neurodegenerative diseases^58,59^. Recent research has even shown that healthy mitochondria transplantation in a conditional *DNM1L* KO model alleviated cerebellar ataxia, suggesting mitochondrial homeostasis restoration as a viable treatment strategy^60^. In addition, bezafibrate has been reported to improve mitochondrial functions in patient fibroblasts harboring a *DNM1L* mutation, further supporting its therapeutic relevance^61^. The perinatal window of reversibility holds great clinical significance as it opens the door to a broader range of therapeutic interventions. Prophylactic treatment during the susceptible perinatal period has shown its efficiency in preventing long-term neurological defects such as epilepsy^62^. Therefore, our findings provide valuable insights into future clinical strategies for early onset and rapidly progressing *DNM1L*-related neurodevelopmental disorders.

Our results and interpretation have several limitations. First, we did not characterize the complete spectrum of DAE pathology, particularly the seizure activity and behavioral phenotypes that represent critical clinical manifestations in patients, such as intractable epilepsy. This limitation stems from constraints in the current experimental models, including the restricted temporal and spatial expression pattern of *DNM1L* variants in the IUE mouse model and the relative immaturity of the cellular system. Consequently, despite the observed molecular and histological rescue, these findings do not yet establish that the same therapeutic strategy would achieve symptomatic improvement in clinical settings. Besides, the efficacy of mitochondrial biogenesis enhancement was demonstrated in a limited developmental window and its long-term effects on brain development and function require thorough investigation. Future studies should incorporate improved animal models with comprehensive phenotypic characterization to bridge the gap between our results and clinical translation.

## Supplementary information

Supplementary Information

## Data Availability

The raw RNA sequencing data have been deposited at SRA (PRJNA1282717) and are publicly available as of the date of publication. This Article does not report original code. All information reported in this Article is available from the lead contact upon request.

## Code availability

This Article does not report original code. All information reported in this Article is available from the lead contact upon request.

## References

[1] Waterham, H. R. et al. A lethal defect of mitochondrial and peroxisomal fission. *N. Engl. J. Med.* **356**, 1736–1741 (2007). 17460227 10.1056/NEJMoa064436

[2] Chang, C. R. et al. A lethal de novo mutation in the middle domain of the dynamin-related GTPase Drp1 impairs higher order assembly and mitochondrial division. *J. Biol. Chem.* **285**, 32494–32503 (2010). 20696759 10.1074/jbc.M110.142430 PMC2952251

[3] Chao, Y. H. et al. Missense variants in the middle domain of DNM1L in cases of infantile encephalopathy alter peroxisomes and mitochondria when assayed in *Drosophila*. *Hum. Mol. Genet.* **25**, 1846–1856 (2016). 26931468 10.1093/hmg/ddw059 PMC5007591

[4] Nasca, A. et al. Biallelic mutations in DNM1L are associated with a slowly progressive infantile encephalopathy. *Hum. Mutat.* **37**, 898–903 (2016). 27328748 10.1002/humu.23033 PMC5108486

[5] Vanstone, J. R. et al. DNM1L-related mitochondrial fission defect presenting as refractory epilepsy. *Eur. J. Hum. Genet.* **24**, 1084–1088 (2016). 26604000 10.1038/ejhg.2015.243 PMC5070894

[6] Yoon, G. et al. Lethal disorder of mitochondrial fission caused by mutations in DNM1L. *J. Pediatr.* **171**, 313–316 e311–e312 (2016). 26825290 10.1016/j.jpeds.2015.12.060

[7] Zaha, K. et al. DNM1L-related encephalopathy in infancy with Leigh syndrome-like phenotype and suppression-burst. *Clin. Genet.* **90**, 472–474 (2016). 27301544 10.1111/cge.12805

[8] Gerber, S. et al. Mutations in DNM1L, as in OPA1, result in dominant optic atrophy despite opposite effects on mitochondrial fusion and fission. *Brain* **140**, 2586–2596 (2017). 28969390 10.1093/brain/awx219

[9] Hogarth, K. A., Costford, S. R., Yoon, G., Sondheimer, N. & Maynes, J. T. DNM1L variant alters baseline mitochondrial function and response to stress in a patient with severe neurological dysfunction. *Biochem. Genet.* **56**, 56–77 (2018). 29110115 10.1007/s10528-017-9829-2

[10] Assia Batzir, N. et al. De novo missense variant in the GTPase effector domain (GED) of DNM1L leads to static encephalopathy and seizures. *Cold Spring Harb. Mol. Case Stud.*10.1101/mcs.a003673 (2019). 10.1101/mcs.a003673 PMC6549558 30850373

[11] Longo, F. et al. Impaired turnover of hyperfused mitochondria in severe axonal neuropathy due to a novel DRP1 mutation. *Hum. Mol. Genet.* **29**, 177–188 (2020). 31868880 10.1093/hmg/ddz211

[12] Liu, X. et al. DNM1L-related mitochondrial fission defects presenting as encephalopathy: a case report and literature review. *Front. Pediatr.* **9**, 626657 (2021). 34307245 10.3389/fped.2021.626657 PMC8295552

[13] Nolden, K. A. et al. Novel DNM1L variants impair mitochondrial dynamics through divergent mechanisms. *Life Sci. Alliance*10.26508/lsa.202101284 (2022). 10.26508/lsa.202101284 PMC9354038 35914810

[14] Zhang, Z. et al. A novel variant of DNM1L expanding the clinical phenotypic spectrum: a case report and literature review. *BMC Pediatr.* **24**, 104 (2024). 38341530 10.1186/s12887-023-04442-y PMC10858475

[15] Nunnari, J. & Suomalainen, A. Mitochondria: in sickness and in health. *Cell* **148**, 1145–1159 (2012). 22424226 10.1016/j.cell.2012.02.035 PMC5381524

[16] Bauer, B. L., Rochon, K., Liu, J. C., Ramachandran, R. & Mears, J. A. Disease-associated mutations in Drp1 have fundamentally different effects on the mitochondrial fission machinery. *Hum. Mol. Genet.* **32**, 1975–1987 (2023). 36795043 10.1093/hmg/ddad029 PMC10244223

[17] Robertson, G. L. et al. DRP1 mutations associated with EMPF1 encephalopathy alter mitochondrial membrane potential and metabolic programs. *J. Cell Sci.*10.1242/jcs.260370 (2023). 10.1242/jcs.260370 PMC10657212 36763487

[18] Ishihara, N. et al. Mitochondrial fission factor Drp1 is essential for embryonic development and synapse formation in mice. *Nat. Cell Biol.* **11**, 958–966 (2009). 19578372 10.1038/ncb1907

[19] Wakabayashi, J. et al. The dynamin-related GTPase Drp1 is required for embryonic and brain development in mice. *J. Cell Biol.* **186**, 805–816 (2009). 19752021 10.1083/jcb.200903065 PMC2753156

[20] Wang, W. et al. Parkinson’s disease-associated mutant VPS35 causes mitochondrial dysfunction by recycling DLP1 complexes. *Nat. Med.* **22**, 54–63 (2016). 26618722 10.1038/nm.3983 PMC4826611

[21] Fowler, P. C., Byrne, D. J., Blackstone, C. & O’Sullivan, N. C. Loss of the mitochondrial fission GTPase Drp1 contributes to neurodegeneration in a *Drosophila* model of hereditary spastic paraplegia. *Brain Sci.*10.3390/brainsci10090646 (2020). 10.3390/brainsci10090646 PMC7564485 32957716

[22] Chen, W., Zhao, H. & Li, Y. Mitochondrial dynamics in health and disease: mechanisms and potential targets. *Signal Transduct. Target Ther.* **8**, 333 (2023). 37669960 10.1038/s41392-023-01547-9 PMC10480456

[23] Verrigni, D. et al. Clinical-genetic features and peculiar muscle histopathology in infantile DNM1L-related mitochondrial epileptic encephalopathy. *Hum. Mutat.* **40**, 601–618 (2019). 30801875 10.1002/humu.23729

[24] Magistrati, M. et al. De novo DNM1L pathogenic variant associated with lethal encephalocardiomyopathy-case report and literature review. *Int. J. Mol. Sci.*10.3390/ijms26020846 (2025). 10.3390/ijms26020846 PMC11765995 39859560

[25] Kim, Y. E. et al. Reversibility and developmental neuropathology of linear nevus sebaceous syndrome caused by dysregulation of the RAS pathway. *Cell Rep.* **42**, 112003 (2023). 36641749 10.1016/j.celrep.2023.112003

[26] Bastin, J., Aubey, F., Rotig, A., Munnich, A. & Djouadi, F. Activation of peroxisome proliferator-activated receptor pathway stimulates the mitochondrial respiratory chain and can correct deficiencies in patients’ cells lacking its components. *J. Clin. Endocrinol. Metab.* **93**, 1433–1441 (2008). 18211970 10.1210/jc.2007-1701

[27] Lund, M. et al. Bezafibrate activation of PPAR drives disturbances in mitochondrial redox bioenergetics and decreases the viability of cells from patients with VLCAD deficiency. *Biochim. Biophys. Acta Mol. Basis Dis.* **1867**, 166100 (2021). 33549744 10.1016/j.bbadis.2021.166100

[28] da Rosa-Junior, N. T. et al. Bezafibrate in vivo administration prevents 3-methylglutaric acid-induced impairment of redox status, mitochondrial biogenesis, and neural injury in brain of developing rats. *Neurotox. Res.* **35**, 809–822 (2019). 30850947 10.1007/s12640-019-00019-9

[29] Dehairs, J., Talebi, A., Cherifi, Y. & Swinnen, J. V. CRISP-ID: decoding CRISPR mediated indels by Sanger sequencing. *Sci. Rep.* **6**, 28973 (2016). 27363488 10.1038/srep28973 PMC4929496

[30] Lin, H. J., Wang, X., Shaffer, K. M., Sasaki, C. Y. & Ma, W. Characterization of H_2_O_2_-induced acute apoptosis in cultured neural stem/progenitor cells. *FEBS Lett.* **570**, 102–106 (2004). 15251448 10.1016/j.febslet.2004.06.019

[31] Nagashima, S. et al. Golgi-derived PI(4)P-containing vesicles drive late steps of mitochondrial division. *Science* **367**, 1366–1371 (2020). 32193326 10.1126/science.aax6089

[32] Friedman, J. R. et al. ER tubules mark sites of mitochondrial division. *Science* **334**, 358–362 (2011). 21885730 10.1126/science.1207385 PMC3366560

[33] Mears, J. A. et al. Conformational changes in Dnm1 support a contractile mechanism for mitochondrial fission. *Nat. Struct. Mol. Biol.* **18**, 20–26 (2011). 21170049 10.1038/nsmb.1949 PMC3059246

[34] Frohlich, C. et al. Structural insights into oligomerization and mitochondrial remodelling of dynamin 1-like protein. *EMBO J.* **32**, 1280–1292 (2013). 23584531 10.1038/emboj.2013.74 PMC3642683

[35] Kalia, R. et al. Structural basis of mitochondrial receptor binding and constriction by DRP1. *Nature* **558**, 401–405 (2018). 29899447 10.1038/s41586-018-0211-2 PMC6120343

[36] Schymkowitz, J. et al. The FoldX web server: an online force field. *Nucleic Acids Res.* **33**, W382–W388 (2005). 15980494 10.1093/nar/gki387 PMC1160148

[37] Toma, K. & Hanashima, C. Switching modes in corticogenesis: mechanisms of neuronal subtype transitions and integration in the cerebral cortex. *Front. Neurosci.* **9**, 274 (2015). 26321900 10.3389/fnins.2015.00274 PMC4531338

[38] Chen, F., Maher, B. J. & LoTurco, J. J. PiggyBac transposon-mediated cellular transgenesis in mammalian forebrain by in utero electroporation. *Cold Spring Harb. Protoc.* **2014**, 741–749 (2014). 24987137 10.1101/pdb.prot073650

[39] Galvis-Montes, D. S. et al. Highly dynamic inflammatory and excitability transcriptional profiles in hippocampal CA1 following status epilepticus. *Sci. Rep.* **13**, 22187 (2023). 38092829 10.1038/s41598-023-49310-y PMC10719343

[40] Kim, T. W. et al. TNF-NF-kappaB-p53 axis restricts in vivo survival of hPSC-derived dopamine neurons. *Cell* **187**, 3671–3689 (2024). 38866017 10.1016/j.cell.2024.05.030 PMC11641762

[41] Ramanujan, A., Li, Z., Ma, Y., Lin, Z. & Ibanez, C. F. RhoGDI phosphorylation by PKC promotes its interaction with death receptor p75(NTR) to gate axon growth and neuron survival. *EMBO Rep.* **25**, 1490–1512 (2024). 38253689 10.1038/s44319-024-00064-2 PMC10933337

[42] Cahill, K. M., Huo, Z., Tseng, G. C., Logan, R. W. & Seney, M. L. Improved identification of concordant and discordant gene expression signatures using an updated rank-rank hypergeometric overlap approach. *Sci. Rep.* **8**, 9588 (2018). 29942049 10.1038/s41598-018-27903-2 PMC6018631

[43] Keenan, A. B. et al. ChEA3: transcription factor enrichment analysis by orthogonal omics integration. *Nucleic Acids Res.* **47**, W212–W224 (2019). 31114921 10.1093/nar/gkz446 PMC6602523

[44] Fabregat, A. et al. Reactome pathway analysis: a high-performance in-memory approach. *BMC Bioinformatics* **18**, 142 (2017). 28249561 10.1186/s12859-017-1559-2 PMC5333408

[45] Cardanho-Ramos, C. & Morais, V. A. Mitochondrial biogenesis in neurons: how and where. *Int. J. Mol. Sci.*10.3390/ijms222313059 (2021). 10.3390/ijms222313059 PMC8657637 34884861

[46] Liu, L., Li, Y., Chen, G. & Chen, Q. Crosstalk between mitochondrial biogenesis and mitophagy to maintain mitochondrial homeostasis. *J. Biomed. Sci.* **30**, 86 (2023). 37821940 10.1186/s12929-023-00975-7 PMC10568841

[47] Valero, T. Mitochondrial biogenesis: pharmacological approaches. *Curr. Pharm. Des.* **20**, 5507–5509 (2014). 24606795 10.2174/138161282035140911142118

[48] Eid, T. Progressive neuronal loss in epilepsy - A long-standing conundrum finally resolved?. *Epilepsy Curr.* **21**, 366–368 (2021). 34924838 10.1177/15357597211030385 PMC8655263

[49] Mathern, G. W. & Bertram, E. H. 3rd. Recurrent limbic seizures do not cause hippocampal neuronal loss: a prolonged laboratory study. *Neurobiol. Dis.* **148**, 105183 (2021). 33207277 10.1016/j.nbd.2020.105183 PMC7855788

[50] Wangler, M. F. et al. The expanding neurological phenotype of DNM1L-related disorders. *Brain* **141**, e28 (2018). 29529134 10.1093/brain/awy024 PMC11505533

[51] Blanquie, O. et al. Electrical activity controls area-specific expression of neuronal apoptosis in the mouse developing cerebral cortex. e*Life*10.7554/eLife.27696 (2017). 10.7554/eLife.27696 PMC5582867 28826501

[52] Wong, F. K. et al. Pyramidal cell regulation of interneuron survival sculpts cortical networks. *Nature* **557**, 668–673 (2018). 29849154 10.1038/s41586-018-0139-6 PMC6207348

[53] Knaus, L. S. et al. Large neutral amino acid levels tune perinatal neuronal excitability and survival. *Cell* **186**, 1950–1967 (2023). 36996814 10.1016/j.cell.2023.02.037

[54] Moujalled, D., Strasser, A. & Liddell, J. R. Molecular mechanisms of cell death in neurological diseases. *Cell Death Differ.* **28**, 2029–2044 (2021). 34099897 10.1038/s41418-021-00814-y PMC8257776

[55] Choi, S. Y. et al. Drp1-mediated mitochondrial dynamics and survival of developing chick motoneurons during the period of normal programmed cell death. *FASEB J.* **27**, 51–62 (2013). 22997225 10.1096/fj.12-211920 PMC3528306

[56] Shields, L. Y. et al. Dynamin-related protein 1 is required for normal mitochondrial bioenergetic and synaptic function in CA1 hippocampal neurons. *Cell Death Dis.* **6**, e1725 (2015). 25880092 10.1038/cddis.2015.94 PMC4650558

[57] Motori, E. et al. Neuronal metabolic rewiring promotes resilience to neurodegeneration caused by mitochondrial dysfunction. *Sci. Adv.* **6**, eaba8271 (2020). 32923630 10.1126/sciadv.aba8271 PMC7455195

[58] Steele, H. et al. Metabolic effects of bezafibrate in mitochondrial disease. *EMBO Mol. Med.* **12**, e11589 (2020). 32107855 10.15252/emmm.201911589 PMC7059007

[59] Inak, G. et al. Defective metabolic programming impairs early neuronal morphogenesis in neural cultures and an organoid model of Leigh syndrome. *Nat. Commun.* **12**, 1929 (2021). 33771987 10.1038/s41467-021-22117-z PMC7997884

[60] Li, S. J. et al. Mitochondria transplantation transiently rescues cerebellar neurodegeneration improving mitochondrial function and reducing mitophagy in mice. *Nat. Commun.* **16**, 2839 (2025). 40121210 10.1038/s41467-025-58189-4 PMC11929859

[61] Douiev, L., Sheffer, R., Horvath, G. & Saada, A. Bezafibrate improves mitochondrial fission and function in DNM1L-deficient patient cells. *Cells*10.3390/cells9020301 (2020). 10.3390/cells9020301 PMC7072316 32012656

[62] Marguet, S. L. et al. Treatment during a vulnerable developmental period rescues a genetic epilepsy. *Nat. Med.* **21**, 1436–1444 (2015). 26594844 10.1038/nm.3987

