# Supplementary material for: Reversibility and therapeutic feasibility of DNM1L-associated neurodevelopmental disorders
Source: Exp Mol Med. 2026 Mar 5;58(3):755–67. doi: 10.1038/s12276-026-01660-z (PMC13049093; doi:10.1038/s12276-026-01660-z)
Supplement: Supplementary file 1 — Supplementary Information [file 12276_2026_1660_MOESM1_ESM.pdf]

Supplementary Information **The PDF file**

includes:

Supplementary Figures 1–9, Supplementary Tables 1 and 2, and supplemental references

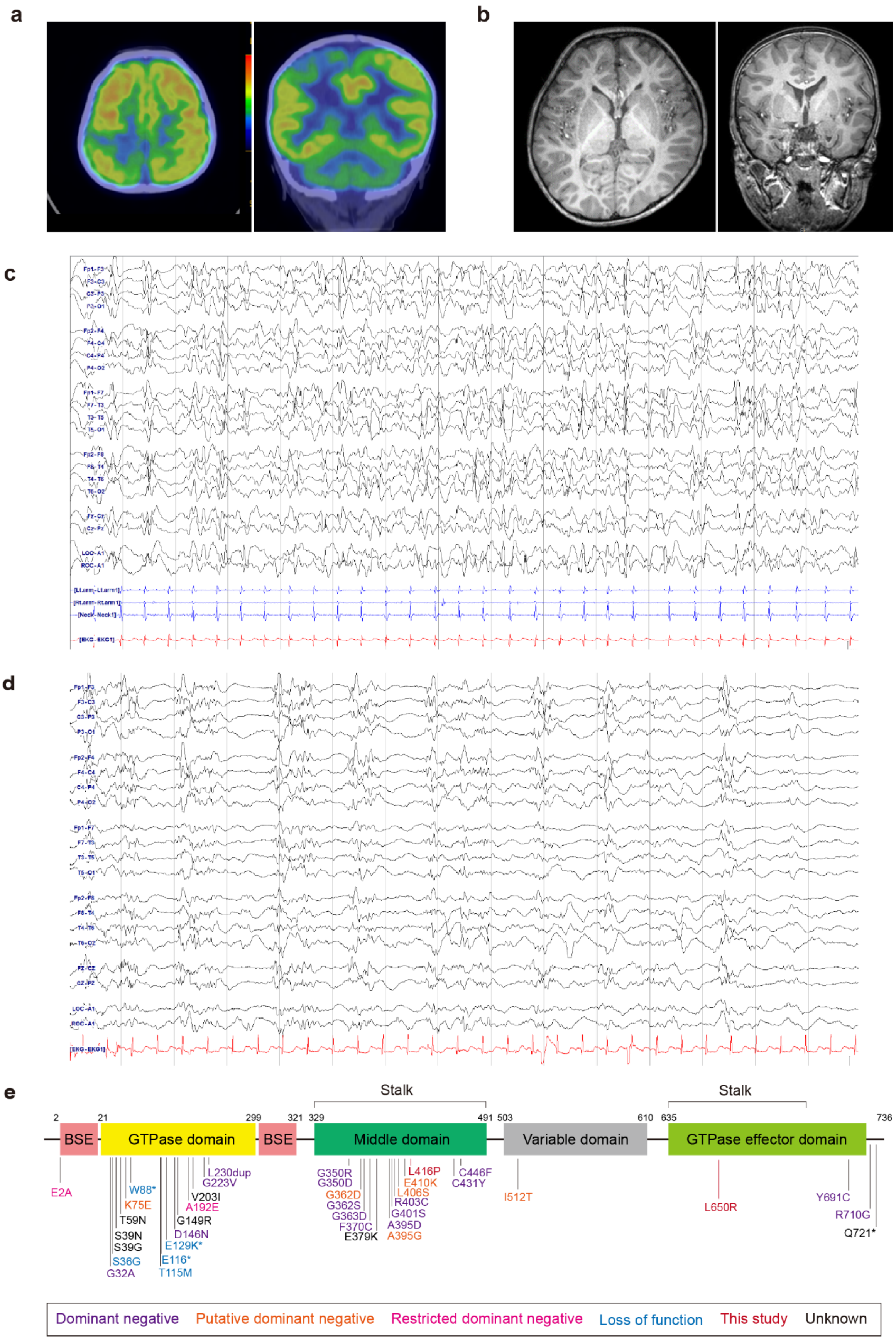
a
b
c
d
e
Stalk
Stalk
2
21
299
321
329
491
503
610
635
736
BSE
GTPase domain
BSE
Middle domain
Variable domain
GTPase effector domain
E2A
W88*
K75E
T59N
S39N
S39G
S36G
G32A
L230dup
G223V
V203I
A192E
G149R
D146N
E129K*
E116*
T115M
G350R
G350D
G362D
G362S
G363D
F370C
E379K
L416P
E410K
L406S
R403C
G401S
A395D
A395G
C446F
C431Y
I512T
L650R
Y691C
R710G
Q721*
Dominant negative
Putative dominant negative
Restricted dominant negative
Loss of function
This study
Unknown

Supplementary Fig. 1 Symptom presentation of DAE patients.
a. FDG-PET brain scans of patient 1 taken at 1 year and 8 months. **b.** T1 axial view MRI of patient 2 taken at the age of 11 months shows no structural abnormality. **c.** EEG of patient 1 taken at the age of 3 years and 8 months. **d.** EEG of patient 2 taken at the age of 4 years and 5 months. **e.** *DNM1L* mutations associated with human disease and their effects on gene function. Domains of DNM1L are labeled as indicated, with stalk domains shown above. Purple: dominant negative heterozygous mutations. Orange: putative dominant negative heterozygous mutations. Pink: heterozygous mutations associated with isolated optic atrophy reported by Gerber *et al*[1]. Dark blue: compound heterozygous mutations resulting in loss of function. Red: *de novo* heterozygous mutations reported in this study. Black: unknown functionality.

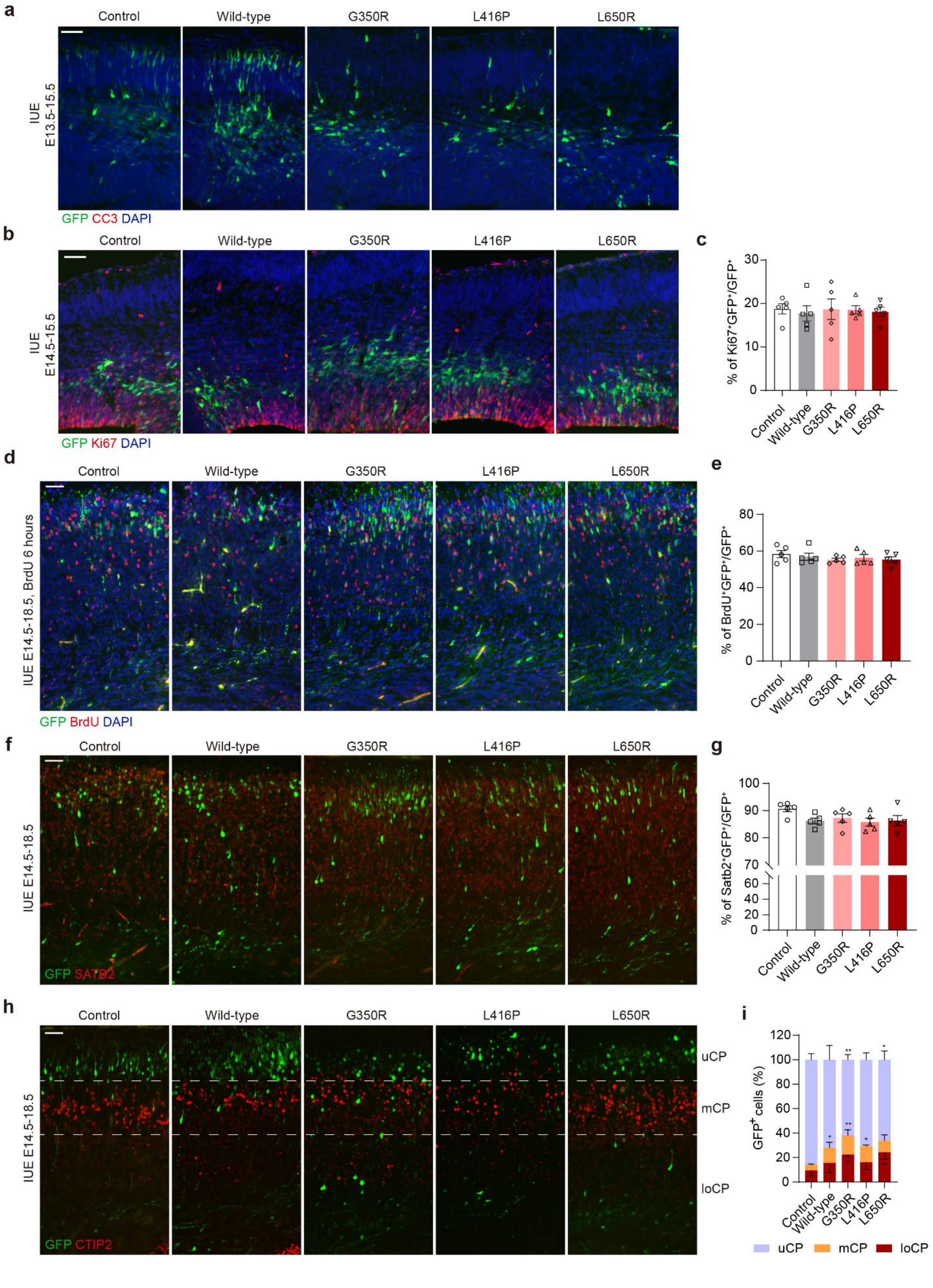
a
Control
Wild-type
G350R
L416P
L650R
IUE
E13.5-15.5
GFP CC3 DAPI
b
IUE
E14.5-15.5
GFP Ki67 DAPI
c
% of Ki67+GFP+/GFP+
d
IUE E14.5-18.5, BrdU 6 hours
GFP BrdU DAPI
e
% of BrdU+GFP+/GFP+
f
IUE E14.5-18.5
GFP SATB2
g
% of Satb2+GFP+/GFP+
h
IUE E14.5-18.5
GFP CTIP2
uCP
mCP
loCP
i
GFP+ cells (%)

Supplementary Fig. 2 Prenatal developmental phenotypes of *DNM1L* mutations.

a. Representative images of E15.5 mouse brain slices electroporated at E13.5 with vectors containing each *DNM1L* variant. No CC3 positive signal was detected. Red, CC3; green, GFP; blue, DAPI. Scale bar, 50 µm. **b, c** Representative images and quantifications of E15.5 mouse brain slices electroporated at E14.5 with vectors containing each *DNM1L* variant. Red, Ki67; green, GFP; blue, DAPI. Scale bar, 50 µm. $n = 5$. **d, e.** Representative images of E18.5 mouse brain slices electroporated at E14.5 with vectors containing each *DNM1L* variant, administered with BrdU intraperitoneally 6 hours after surgery. Red, BrdU; green, GFP; blue, DAPI. Scale bar, 50 µm. $BrdU^+$ cells among GFP+ cells in E18.5 mouse brain slices were quantified. $n = 5$. **f, g.** Representative images of E18.5 mouse brain slices electroporated at E14.5 with vectors containing each *DNM1L* variant. Red, Satb2; green, GFP; blue, DAPI. Scale bar, 50 µm. $Satb2^+$ cells among GFP^+ cells in E18.5 mouse brain slices were quantified. $n = 5$.

h, i. Representative images of E18.5 mouse brain slices immunostained with Ctip2. Red, Ctip2; green, GFP; blue, DAPI. Scale bar, 50 µm. Neuronal migration was characterized by the percentages of GFP^+ cells in each layer. $n = 5$. Bar plot indicates mean ± SEM. Statistical significance is determined by the Kruskal-Wallis test with Dunn's post hoc test for **c** and **e**, one-way ANOVA with Tukey's post hoc test for **g**, and two-way ANOVA with Dunnett's post hoc test for **i**. $^*p < 0.05$, $^{**}p < 0.01$. loCP, lower cortical plate; mCP, middle cortical plate; uCP, upper cortical plate.

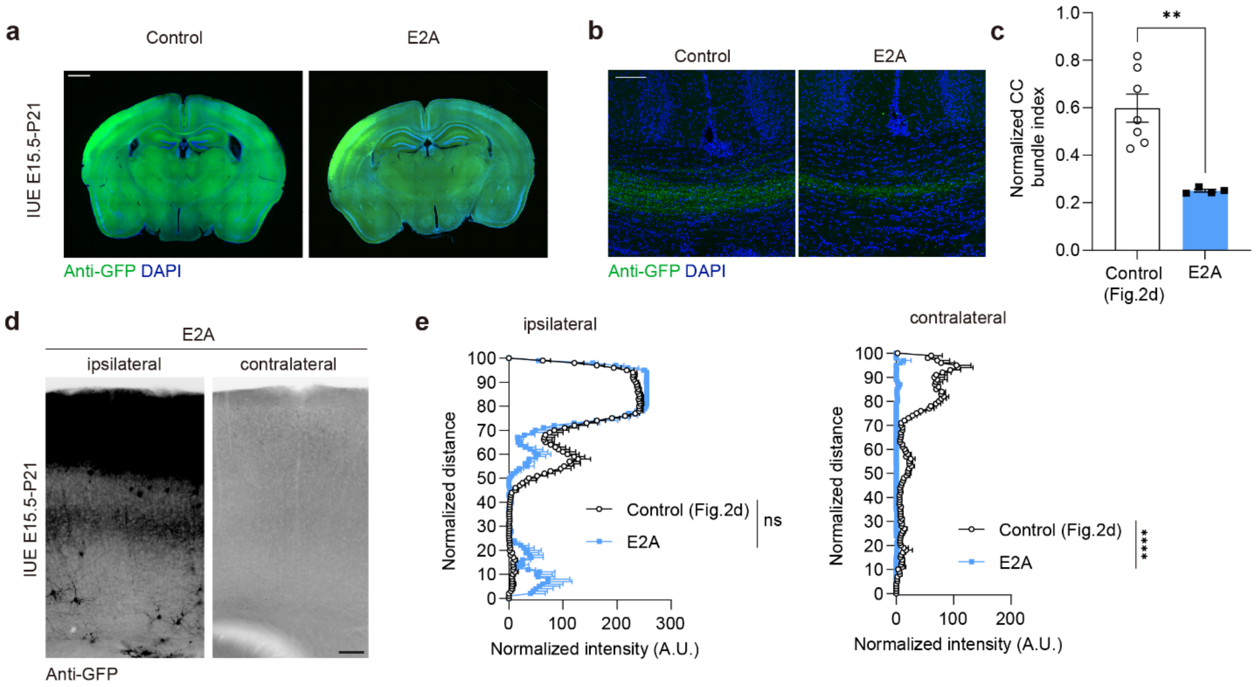

Supplementary Fig. 3 Neurodevelopmental defects caused by *DNM1L^{E2A}*.

a. Representative images of whole brain slices electroporated with each variant. Green, GFP; blue, DAPI. Scale bar, 1 mm. **b, c.** Representative confocal z-stack images and quantifications of 35 µm sections of a P21 mouse brain CC. Control, $n = 7$; E2A, $n = 4$. GFP; blue, DAPI. Scale bar, 100 µm. **d, e.** Representative images and quantifications of ipsilateral/contralateral cortical plates electroporated with *DNM1L^{E2A}*. $n = 4$. Scale bar, 200 µm. Bar plot indicates mean ± SEM. Statistical significance is determined by the Mann-Whitney U test for **c** and two-way ANOVA with Bonferroni's post hoc test for **e**. $^{**}p < 0.01$; $^{****}p < 0.0001$; ns, not significant.

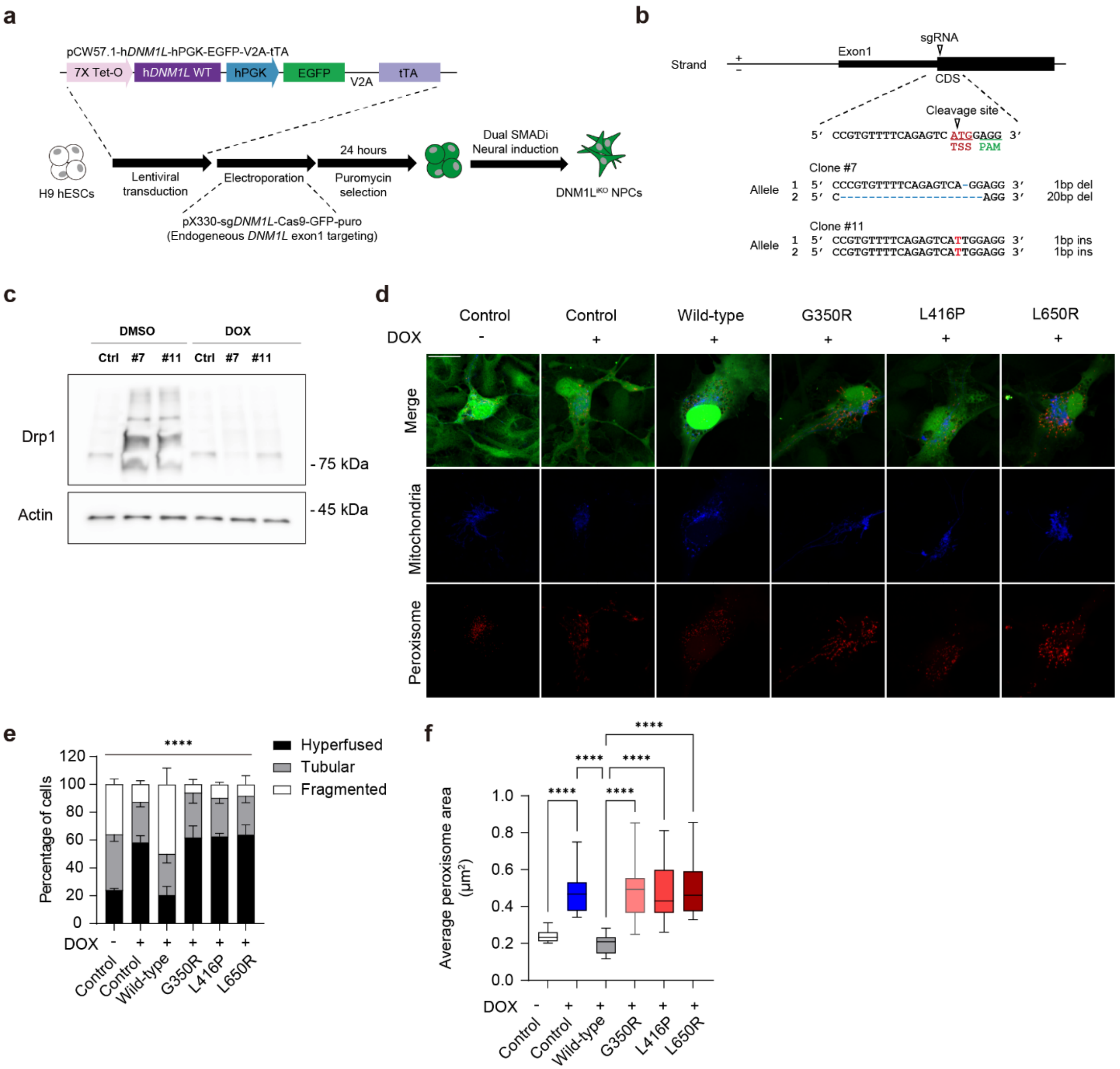
a
pCW57.1-hDNM1L-hPGK-EGFP-V2A-tTA
7X Tet-O
hDNM1L WT
hPGK
EGFP
V2A
tTA
H9 hESCs
Lentiviral transduction
Electroporation
24 hours
Puromycin selection
Dual SMADi Neural induction
DNM1L^{iKO} NPCs
pX330-sgDNM1L-Cas9-GFP-puro
(Endogeneous DNM1L exon1 targeting)
b
sgRNA
Strand
+
−
Exon1
CDS
Cleavage site
5′ CCGTGTTTTCAGAGTC ATG GAGG 3′
TSS
PAM
Clone #7
Allele 1 5′ CCCGTGTTTTCAGAGTCA-GGAGG 3′ 1bp del
2 5′ C-------------------AGG 3′ 20bp del
Clone #11
Allele 1 5′ CCGTGTTTTCAGAGTCATTGGAGG 3′ 1bp ins
2 5′ CCGTGTTTTCAGAGTCATTGGAGG 3′ 1bp ins
c
DMSO
DOX
Ctrl #7 #11 Ctrl #7 #11
Drp1
Actin
75 kDa
45 kDa
d
Control
Control
Wild-type
G350R
L416P
L650R
DOX
-
+
+
+
+
+
Merge
Mitochondria
Peroxisome
e
****
Hyperfused
Tubular
Fragmented
Percentage of cells
120
100
80
60
40
20
0
DOX - + + + + +
Control
Control
Wild-type
G350R
L416P
L650R
f
****
****
****
****
****
Average peroxisome area (μm²)
1.0
0.8
0.6
0.4
0.2
0.0
DOX - + + + + +
Control
Control
Wild-type
G350R
L416P
L650R

Supplementary Fig. 4 Characterization of DNML^{iKO} hESC-derived neuronal cell line.
a. Schematic working flow for DNML^{iKO} hESC-derived neuronal cell line establishment. **b.** Schematic illustration of the strategy for targeting the endogenous *DNM1L* allele in hESCs using CRISPR/Cas9 genome editing. Sequence information of the targeted allele from the established two clones is shown. **c.** Western blot analysis of DRP1 levels in control and DNM1L^{iKO} NPCs treated with DMSO or DOX. Actin is used as a loading control. **d.** Confocal microscopy images showing the impact of wild-type and mutant DNM1L expression on mitochondrial and peroxisomal morphology in NPCs; cells were treated with DOX to induce *DNM1L* knockout. Blue, mitochondria; red, peroxisomes; green, GFP. Scale bar, 20 µm. **e.** Quantification of mitochondrial morphology in NPCs expressing different *DNM1L* variants, categorized into hyperfused, tubular, and fragmented. n = 3 with at least 60 cells in each condition analyzed for an independent experiment. **f.** Quantification of the average peroxisome area in NPCs expressing each *DNM1L* variant, with or without DOX treatment. Control + DMSO, n = 20; control + DOX, n = 15; wild-type + DOX, n = 17; G350R + DOX, n = 15; L416P + DOX, n = 15; L650R + DOX, n = 16. Bar graphs indicate mean ± SEM. Statistical significance is determined by two-way ANOVA with Tukey's post hoc test for **e** and the Kruskal-Wallis test with Dunn's post hoc test for **f**. ****p < 0.0001.

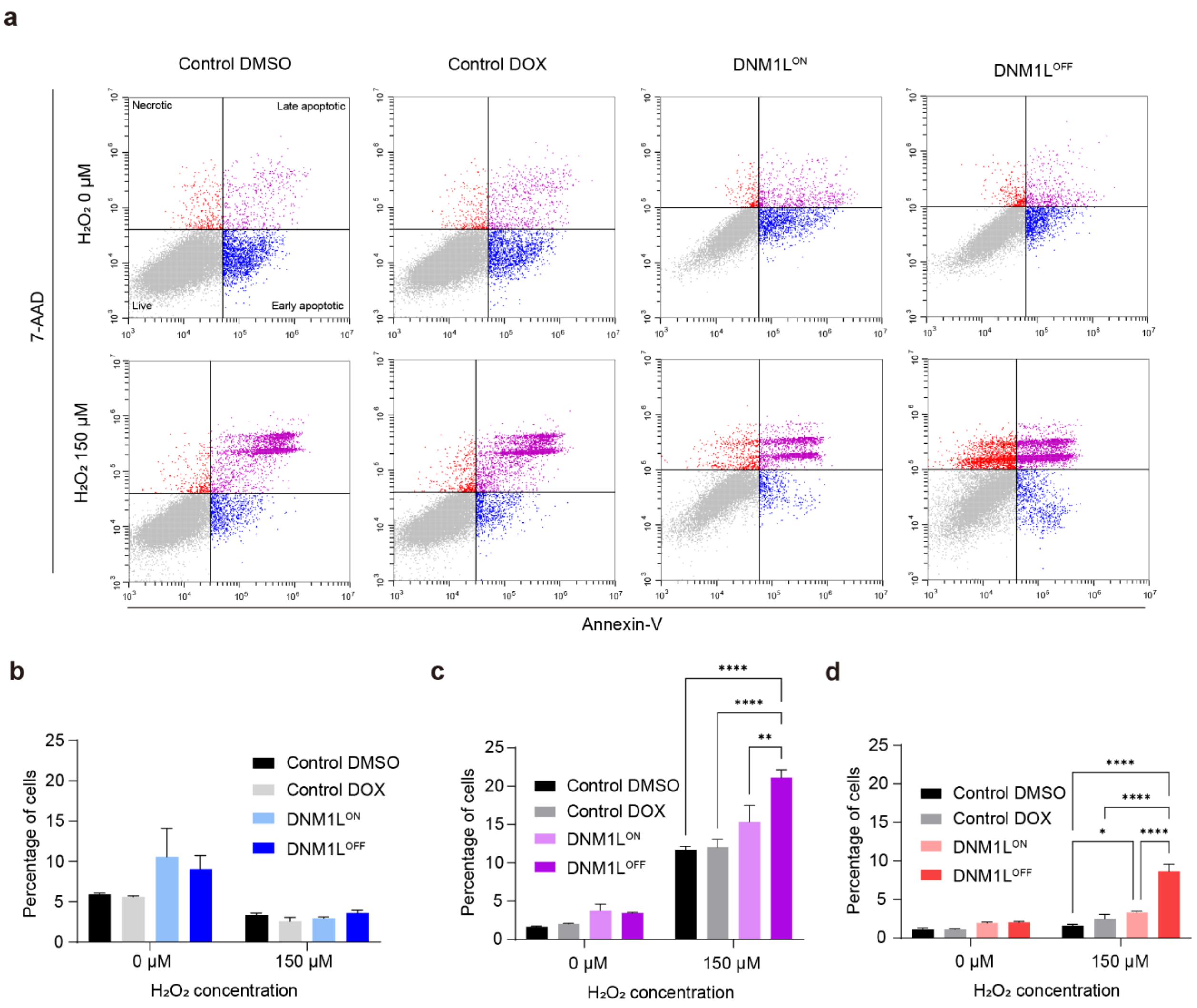

Supplementary Fig. 5 Cell death phenotypes of DNM1L^{iKO} cells upon oxidative stress.
a. Representative flow cytometry dot plots displaying Annexin V and 7-AAD staining in control and DNM1L^{iKO} NPCs treated with DMSO or DOX and subjected to oxidative stress using two concentrations of H_2O_2 (0 µM and 150 µM). Cells are categorized into live, early apoptotic, late apoptotic, and necrotic based on their staining patterns. Grey, live; blue, early apoptotic; purple, late apoptotic; red, necrotic. **b-d.** Quantification of early apoptosis (**b**), late apoptosis (**c**), and necrosis (**d**) in each condition. Bar graphs indicate mean ± SEM. n = 3 technical replicates from 3 biological replicates. Statistical significance is determined by two-way ANOVA with Tukey's post hoc test. $*p < 0.05$, $**p < 0.01$, $****p < 0.0001$.

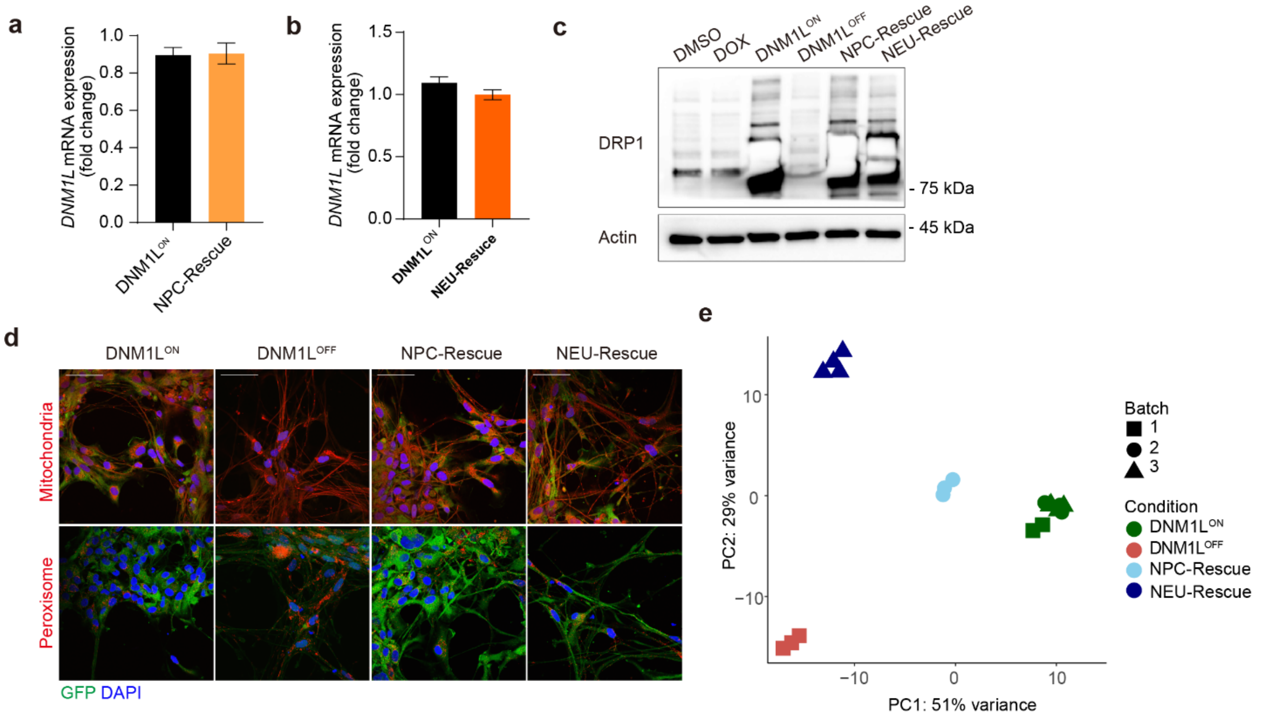

Supplementary Fig. 6 Characterization of rescue conditions in DNM1L^{iKO} differentiated neurons by doxycycline withdrawal.

a, b. qPCR analysis showing relative *DNM1L* mRNA expression in NPC-Rescue (**a**) and NEU-Rescue (**b**) compared to DNM1L^{ON} condition. The *RPLP0* gene was used for normalization control. **c.** Western blot analysis of DRP1 levels in control and DNM1L^{iKO} differentiated neurons with different DOX treatment conditions. Actin is used as a loading control. **d.** Representative confocal microscopy images displaying the mitochondrial and peroxisomal morphology in DNM1L^{iKO} cells with different DOX treatment conditions. Red, mitochondria (Mitotracker CMXRos) or peroxisome (PMP70); green: GFP, blue: DAPI. Scale bar, 20 µm. **e.** PCA plot of batch-corrected RNA sequencing samples across different experimental setups, depicted by respective symbols and colors.

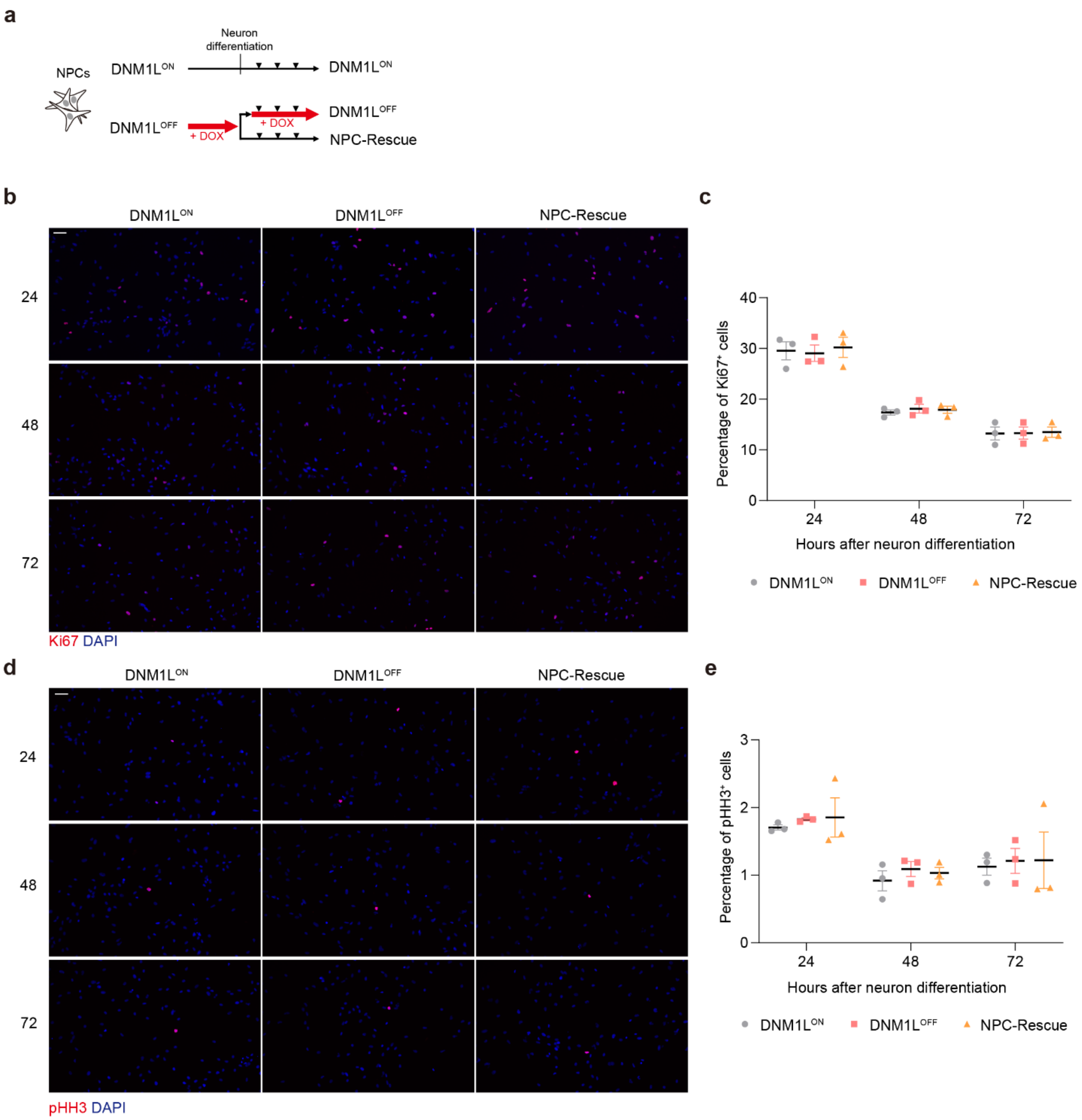

Supplementary Fig. 7 Time-course analysis of cell division during early neuronal induction in DNM1L^{ON}, DNM1L^{OFF}, and NPC-Rescue.

a. Schematic diagram of harvest timeline across conditions. Red arrows indicate DOX treatment during culture, and black arrows indicate the harvest timepoint (24, 48, and 72 hours after neuronal differentiation). **b, c.** Representative images and quantification of Ki67^{+} cells in DNM1L^{ON}, DNM1L^{OFF}, and NPC-Rescue conditions across different timepoints. Red, Ki67; blue, DAPI. Scale bar, 50 µm. **d, e.** Representative images and quantification of pHH3^{+} cells in DNM1L^{ON}, DNM1L^{OFF}, and NPC-Rescue conditions across different timepoints. Red, pHH3; blue, DAPI. Scale bar, 50 µm.

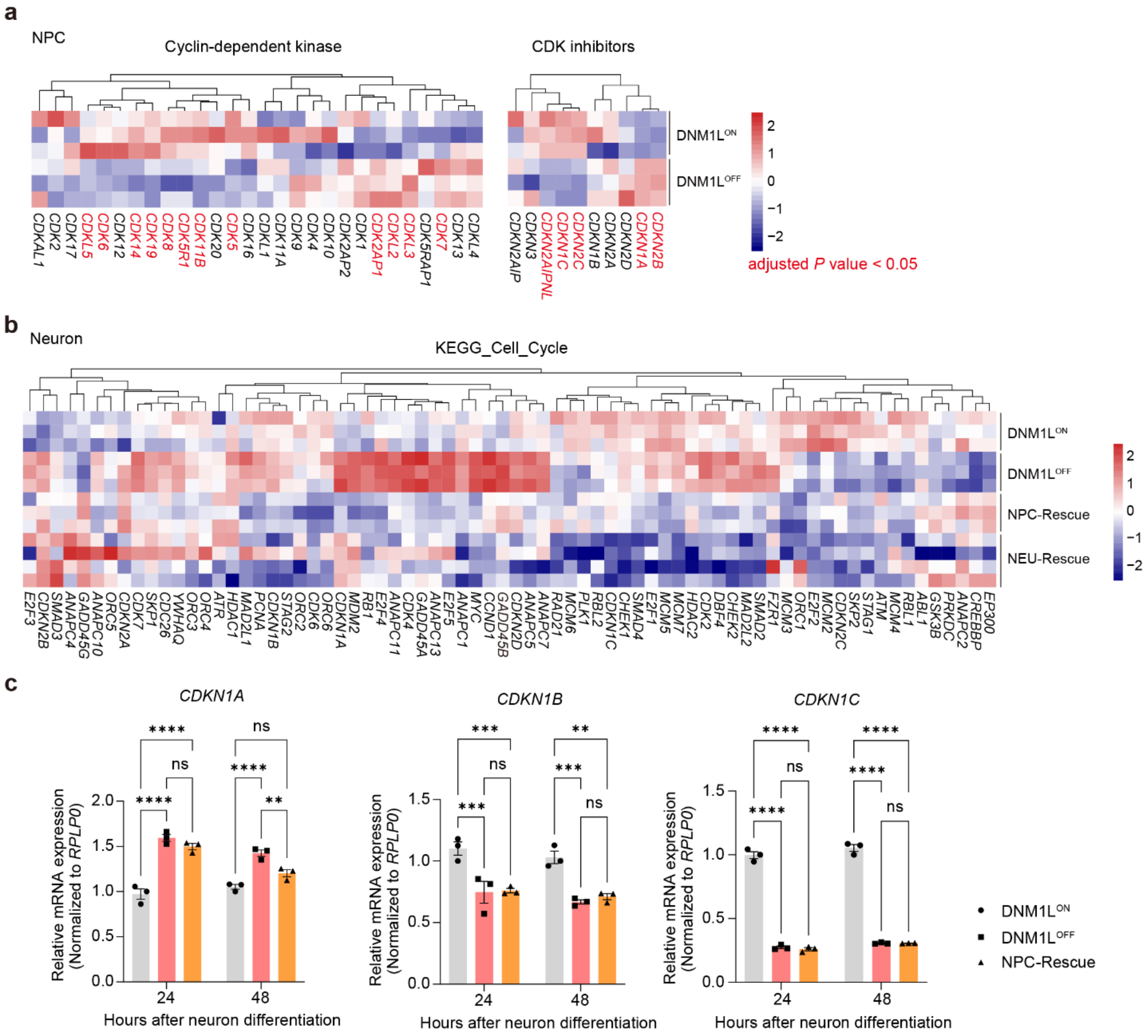

Supplementary Fig. 8 Transcriptomic changes related to cell cycle regulation in DNM1L^{OFF} and rescue conditions.

a. Heatmaps of cell cycle regulation gene expression in DNM1L^{ON} and DNM1L^{OFF} NPCs. The color gradient in the heatmap represents the Z score. Genes with adjusted *P* value smaller than 0.05 were indicated in red. **b**. Heatmaps of cell cycle-related genes from the KEGG database across DNM1L^{ON}, DNM1L^{OFF}, NPC-Rescue, and NEU-Rescue neurons. The color gradient in the heatmap represents the Z score. **c.** qPCR analysis showing relative *CDKN1A*, *CDKN1B*, and *CDKN1C* mRNA expression in DNM1L^{ON}, DNM1L^{OFF}, and NPC-Rescue conditions harvested 24 and 48 hours after neuronal induction. The *RPLP0* gene was used for normalization control. Statistical significance is determined by two-way ANOVA with Tukey's post hoc test. **$p < 0.01$; ***$p < 0.001$; ****$p < 0.0001$.

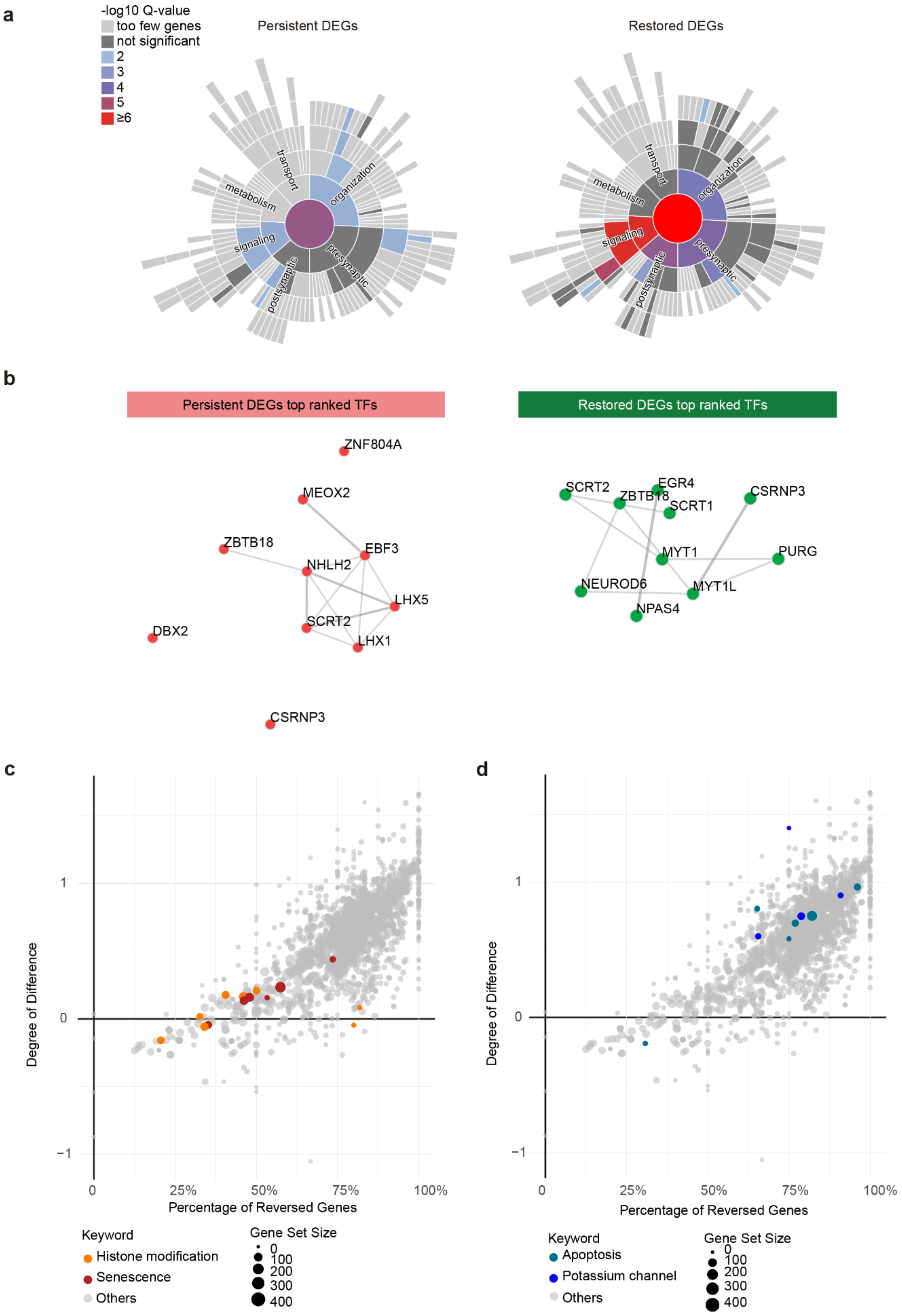
a
-log10 Q-value
too few genes
not significant
2
3
4
5
≥6
Persistent DEGs
Restored DEGs
transport
organization
metabolism
signaling
presynaptic
postsynaptic
b
Persistent DEGs top ranked TFs
Restored DEGs top ranked TFs
ZNF804A
MEOX2
ZBTB18
EBF3
NHLH2
LHX5
SCRT2
LHX1
DBX2
CSRNP3
EGR4
ZBTB18
SCRT1
CSRNP3
MYT1
PURG
NEUROD6
MYT1L
NPAS4
c
d
Degree of Difference
Percentage of Reversed Genes
0
25%
50%
75%
100%
1
0
−1
Keyword
Histone modification
Senescence
Others
Gene Set Size
0
100
200
300
400
Apoptosis
Potassium channel

Supplementary Fig. 9 Transcriptional analysis of persistent and restored DEGs after *DNM1L* expression recovery.

a. SynGO analysis of persistent DEGs (left) and restored DEGs (right). Colors indicate the significance level ($-\log_{10}Q$). **b.** Network diagram of top 10 transcription factors associated with persistent (left, red nodes) and restored (right, green nodes) DEGs predicted by ChEA3. Edges indicate physical interaction supported by literature evidence, directed if supported by ChIP-seq data. **c, d.** Scatter plot illustrating reversibility of Reactome pathways color-coded by keyword with low (**c**) and high (**d**) reversibility. The x-axis is the percentage of reversible genes among the Reactome pathway gene sets, and the y-axis is the difference in the extent of change caused by *DNM1L* dysfunction and under rescue conditions compared to control, expressed as a *Z*-score.

Supplementary Table 1. ***In silico*** **variant effect prediction**

Variant	Polyphen2[2]	Mutation Accessor[3]	VARIETY[4]	EVE[5]	Rhapsody[6]	ESM1b[7]	AlphaMissense[8]
L416P	Probably damaging	High impact	0.993	0.896	0.788 (Deleterious)	-14.21	Pathogenic 0.9999
L650R	Probably damaging	High impact	0.897	0.787	0.764 (Deleterious)	-15.03	Pathogenic 0.9988

Supplementary Table 2. Primers and sgRNA used in this study

	Forward (5'-3')	Reverse (5'-3')
sgRNA_h*DNM1L*	CACCGCCGTGTTTTCAGAGTCATGG	AAACCCATGACTCTGAAAACACGGC
Human *DNM1L* cDNA cloning primers	TTTCTCGAGATGGAGGCGCTAATTCCTGTCATAAACAAGC	TTTGAATTCTCACCAAAGATGAGTCTCCCGGATTTCA
p.E2A mutagenesis primer	TTTCTCGAGATGGCGGCGCTAATTCCTGTCATAAACAAGC	
p.G350R mutagenesis primer	TTGTAACACTATTGAAAGAACTGCAA	TTGCAGTTCTTTCAATAGTGTTACAA
p.L416P mutagenesis primer	ATTTGAGTTACCGGTGAAGCGGCAA	TTGCCGCTTCACCGGTAACTCAAAT
p.L650R mutagenesis primer	GTGAGGTTATTGAACGACGCATTAAATCAT	ATGATTTAATGCGTCGTTCAATAACCTCAC
Human *DNM1L* qPCR primer	GAATTACCTTCAGCTGTATCACGA	TTAACTTGCCATCAGCCTCA
Human *RPLP0* qPCR primer	GGCACCATTGAAATCCTGAG	GACCAGCCCAAAGGAGAAG
Human *CDKN1A* qPCR primer	TGTCCGTCAGAACCCATGC	AAAGTCGAAGTTCCATCGCTC
Human *CDKN1B* qPCR primer	AACGTGCGAGTGTCTAACGG	CCCTCTAGGGGTTTGTGATTCT
Human *CDKN1C* qPCR primer	ACATCCACGATGGAGCGTC	GAAGTCGTAATCCCAGCGGT

Supplementary references

1 Gerber, S. *et al.* Mutations in DNM1L, as in OPA1, result in dominant optic atrophy despite opposite effects on mitochondrial fusion and fission. *Brain* **140**, 2586-2596 (2017). https://doi.org/10.1093/brain/awx219

2 Adzhubei, I., Jordan, D. M. & Sunyaev, S. R. Predicting functional effect of human missense mutations using PolyPhen-2. *Curr Protoc Hum Genet* **Chapter 7**, Unit7 20 (2013). https://doi.org/10.1002/0471142905.hg0720s76

3 Reva, B., Antipin, Y. & Sander, C. Predicting the functional impact of protein mutations: application to cancer genomics. *Nucleic Acids Res* **39**, e118 (2011). https://doi.org/10.1093/nar/gkr407

4 Wu, Y., Li, R., Sun, S., Weile, J. & Roth, F. P. Improved pathogenicity prediction for rare human missense variants. *Am J Hum Genet* **108**, 1891-1906 (2021). https://doi.org/10.1016/j.ajhg.2021.08.012

5 Frazer, J. *et al.* Disease variant prediction with deep generative models of evolutionary data. *Nature* **599**, 91-95 (2021). https://doi.org/10.1038/s41586-021-04043-8

6 Ponzoni, L., Penaherrera, D. A., Oltvai, Z. N. & Bahar, I. Rhapsody: predicting the pathogenicity of human missense variants. *Bioinformatics* **36**, 3084-3092 (2020). https://doi.org/10.1093/bioinformatics/btaa127

7 Brandes, N., Goldman, G., Wang, C. H., Ye, C. J. & Ntranos, V. Genome-wide prediction of disease variant effects with a deep protein language model. *Nat Genet* **55**, 1512-1522 (2023). https://doi.org/10.1038/s41588-023-01465-0

8 Cheng, J. *et al.* Accurate proteome-wide missense variant effect prediction with AlphaMissense. *Science* **381**, eadg7492 (2023). https://doi.org/10.1126/science.adg7492
